# Designed switch from covalent to non-covalent inhibitors of carboxylesterase Notum activity

**DOI:** 10.1016/j.ejmech.2023.115132

**Published:** 2023-05-05

**Authors:** Benjamin N. Atkinson, Nicky J. Willis, Yuguang Zhao, Chandni Patel, Sarah Frew, Kathryn Costelloe, Lorenza Magno, Fredrik Svensson, E. Yvonne Jones, Paul V. Fish

**Affiliations:** a Alzheimer's Research UK UCL Drug Discovery Institute, University College London, The Cruciform Building, Gower Street, London, WC1E 6BT, UK; b Division of Structural Biology, Wellcome Centre for Human Genetics, University of Oxford, The Henry Wellcome Building for Genomic Medicine, Roosevelt Drive, Oxford, OX3 7BN, UK

**Keywords:** Covalent inhibition, Notum inhibitors, Structural biology, Wnt signalling, Acyl indolines

## Abstract

*N*-Acyl indolines **4** are potent, non-covalent Notum inhibitors developed from a covalent virtual screening hit **2a**. The lead compounds were simple to synthesise, achieved excellent potency in a biochemical Notum-OPTS assay and restored Wnt signalling in a cell-based TCF/LEF reporter assay. Multiple high resolution X-ray structures established a common binding mode of these inhibitors with the indoline bound centred in the palmiteolate pocket with key interactions being aromatic stacking and a water mediated hydrogen bond to the oxyanion hole. These *N*-acyl indolines **4** will be useful tools for use in vitro studies to investigate the role of Notum in disease models, especially when paired with a structurally related covalent inhibitor (e.g. **4w** and **2a**). Overall, this study highlights the designed switch from covalent to non-covalent Notum inhibitors and so illustrates a complementary approach for hit generation and target inhibition.

## Highlights

• *N*-Acyl indolines are non-covalent Notum inhibitors developed from a covalent hit.
• Compounds were simple to synthesise with excellent potency in multiple Notum assays.
• Notum-inhibitor binding modes rationalised by X-ray structures.
• These *N*-acyl indolines **4** displayed poor metabolic stability.
• Paired indolines **4w** and **2a** are useful in vitro tools for Notum.

## Graphical abstract

**Figure:**
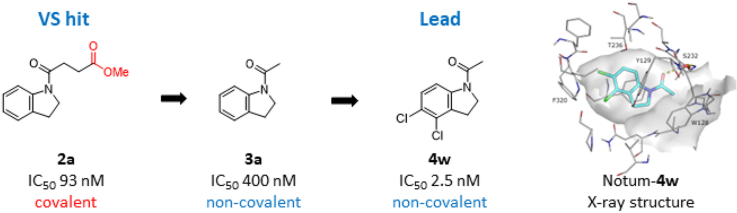

## Abbreviations

ADME: absorption distribution metabolism elimination
CNS: central nervous system
DIPEA: *N*,*N*-diisopropylethylamine
DMF: *N,N*-dimethylformamide
DMSO: dimethylsulfoxide
ER: efflux ratio
HAC: heavy atom count
HLM: human liver microsomes
LE: ligand efficiency
LLE: lipophilic ligand efficiency
MLM: mouse liver microsomes
MoA: mechanism of action
MW: molecular weight
OPTS: trisodium 8-octanoyloxypyrene-1,3,6-trisulfonate
PBS: phosphate buffer saline
PDB: Protein Data Bank
RT: room temperature
SAR: structure activity relationship
SBDD: structure-based drug design
THF: tetrahydrofuran
VS: virtual screening

## 1 Introduction

Carboxylesterase Notum is a serine hydrolase enzyme that catalyses the delipidation of Wnt proteins and so regulates Wnt signalling [1,2]. Wnt signalling plays a key role in embryonic development and adult biology, and has become a drug target of considerable interest for human disease [3]. Notum may also be a valid drug target through its deactivation of Wnt signalling by removal of an essential palmitoleate group needed for the binding of Wnt proteins to the Frizzled cell surface receptors. Inhibition of Notum activity has the potential to restore Wnt signalling tone and may be a therapeutic approach for disease associated with a decrease in Wnt signalling. Recent studies have shown there is a growing understanding of the role Notum plays in human disease such as osteoporosis [4,5] and colorectal cancer [6,7]. Notum has been proposed as a druggable target with reliable screening technologies, multiple structural studies and fit-for-purpose small molecule inhibitors available [8,9].

**Fig. 1:**
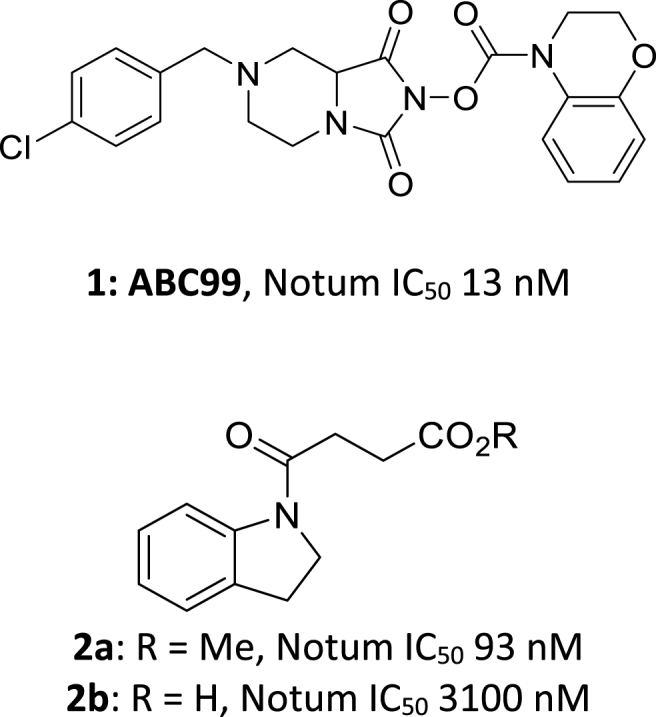
Covalent Notum inhibitors. **ABC99** (**1**) [10] and butyric ester **2a** [22] inactivate Notum through acylation of Ser232.

A noteworthy covalent inhibitor of Notum is carbamate ABC99 (**1**), which is a selective, irreversible inhibitor developed by activity-based protein profiling (Fig. 1) [10]. ABC99 is a valuable research tool that has been used to investigate the link between inhibition of Notum activity with cellular regeneration in aged intestinal epithelium [11] and neurogenesis in the subventricular zone of the brain [12].

The successful development of drugs with a covalent mechanism of action (MoA) has prompted the design of covalent inhibitors for a wide range of proteins through modification of suitable amino acid residues (catalytic or noncatalytic) [[13], [14], [15], [16], [17]]. Covalent inhibitors that react with the serine hydrolase family have long been an important designed modality in the identification of structural and chemical biology tools, and new therapeutics [18,19]. A number of ‘warheads’ have been created that react selectively with the activated, catalytic serine residue in either a reversible or irreversible covalent manner and usually mimic a substrate transition state [18,20].

There has been significant discussion of the risk-benefit of covalent drugs with an evolving understanding that they can offer exceptional potency, selectivity (especially for non-conserved residues), prolonged target engagement, and an approach to treat challenging drug targets [13]. Concerns can arise from overt reactivity of the warheads leading to potentially poor selectivity (conserved residues in families) and toxicity. Hence, to balance these risks, it may be advantageous to develop matched tool inhibitors from the same chemotype offering both covalent and non-covalent MoA, along with an inactive control. This aligned trinity of tools would be a valuable toolbox for use in disease association and target validation studies for a protein of interest.

We have previously described a virtual screen (VS) as a new approach to discover hits as inhibitors of Notum activity [21]. This VS identified several fragment-sized series and singletons that were experimentally validated as nanomolar inhibitors of Notum. One of these hits, butyric ester **2a**, was subsequently shown to act through a covalent mechanism [22]. An X-ray structure of the Notum-**2a** complex showed a covalent adduct had formed between the catalytic Ser232 and the butyric ester (PDB: 7ARG), which was confirmed by mass-spectrometry analysis [22]. The corresponding carboxylic acid **2b** was a weak inhibitor of Notum that did not display covalent inhibition (PDB: 7B37 and 7B3F (S232A)) [22]. This matched pair of inhibitors suggested it could be feasible to switch from covalent to non-covalent MoA within a suitable chemical template.

## 2 Results and discussion

Our objective was to develop potent, non-covalent inhibitors of Notum from this indoline scaffold **2** but without the ester or the acid functional groups. Replacement of the ester would prevent acylation of Ser232 and likely improve stability as esters can be vulnerable to plasma and liver esterases [23]. Removal of the carboxylic acid would promote passive brain permeability and potentially provide a tool suitable for use in exploring Wnt signalling in disease of the CNS [24,25]. In this Article, we describe detailed structure-activity relations (SAR) supported by structure-based drug design (SBDD) that ultimately led to the identification of highly potent, non-covalent inhibitors of Notum activity.

The first round of design was guided by the X-ray crystal structure of Notum-**2b** (PDB: 7B37) that indicated a disordered state of the pendant butyric chain, likely due to a steric clash with Ser232 [22]. A truncated version of **2b** could reduce the flexibility of the chain, avoid the clash with Ser232, and potentially achieve more favourable binding; this gave *N*-acyl indoline **3a** as a prototype inhibitor to test this hypothesis. Screening **3a** in a high-throughput biochemical assay with Notum (81–451C330S) and OPTS as a synthetic fluorescent substrate showed **3a** to be an inhibitor of Notum activity with IC_50_ 0.40 ± 0.11 μM (n = 6) (Table 1). Notably, **3a** is 7-fold more potent than **2b**. Indoline **3a** is a relatively small molecule with moderate lipophilicity (MW 161.2, HAC 12, *c*logP 1.4) and proved to be a highly efficient inhibitor (LE 0.75, LLE 5.0).

**Table 1:** Exploration of *N*-acyl core scaffolds.

Compound	Structure	Notum IC_50_ (μM) a
**2a**	–	0.093 ± 0.020 b
**3a**		0.40 ± 0.11
**3b**		5.3 ± 1.2
**3c**		2.5 ± 1.0
**3d**		2.2 ± 0.4
**3e**		8.0 ± 2.2
**3f**		inactive c
**3g**		>10 d
**3h**		>10 d
**3i**		0.69 ± 0.12
**3j**		3.2 ± 0.66
**3k**		>10 d
**3l**		inactive c
**3m**		2.8 ± 1.8
**3n**		3.6 ± 0.1
**3o**		>10 d

a All values are mean ± s.d. of n = 2–6 experiments quoted to 2 s.f.

b As a covalent inhibitor of Notum, the IC_50_ value will be time-dependant.

c <20% inhibition @ 10 μM.

d 20–50% inhibition @ 10 μM.

**Fig. 2:**
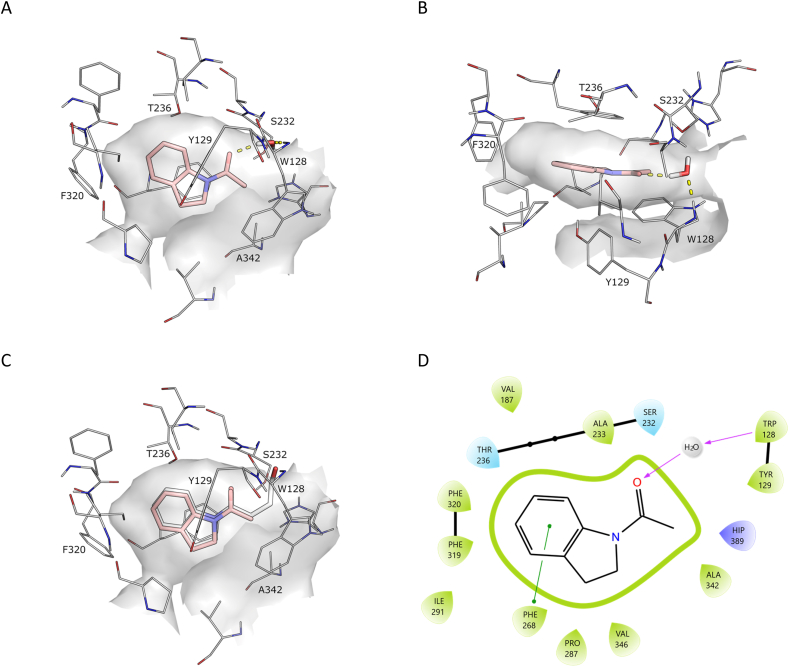
X-ray structure of *N*-acyl indoline **3a** (PDB 8BT8) (salmon). (A) Side view. (B) Top view. (C) Overlay of binding poses of **2a** (white) and **3a** (salmon). (D) Map of key interactions. Select binding site residues shown within 4 Å of their respective ligands. Polar contacts are indicated as dashed lines. Water molecules not directly interacting with the inhibitor have been removed for clarity. The surface of the Notum binding pocket outline (grey) is based on the respective structures. Hydrogens were added and optimised using the Protein Preparation Wizard in Schrödinger Maestro.

The Notum-**3a** X-ray structure revealed clear electron density (Fig. S1). The structure of **3a** showed the inhibitor bound centred in the Notum palmitoleate pocket sandwiched between Phe268 and Tyr129 with the aromatic B-ring stacking against Phe320 (Fig. 2). While not forming any direct hydrogen bonds with Notum, a water molecule (W122) bridges an interaction with the oxyanion hole. Comparison of the relative positions and interactions of **3a** with **2a** showed the binding mode of the acyl indoline motifs was highly conserved in these structures with a near identical overlay. Hence, removal of the butyric ester group from **2a** had not significantly changed or disrupted the binding of the indoline group in the pocket despite deletion of the covalent tether validating the hypothesis that a truncated version of **2b** could achieve a favourable binding mode.

These early findings gave encouragement that our objective was realistic and that **3a** was an attractive hit as a starting point for further optimisation. The successful structure determination of **3a** in Notum provides valuable insight on the binding mode of these non-covalent inhibitors to guide further design in a SBDD enabled manner.

The SARs were aimed at investigating three structural features of **3a**: (1) different scaffolds **3** which mimic the acyl indoline template (Table 1); (2) optimisation of the substituents on preferred scaffolds **4** (Table 2); and (3) fine-tuning of structural features to improve ADME properties **5** (Table 4).

**Table 2:** Substitution patterns on the *N*-acyl indoline core (**4**). .

Compound	R^2^; R^3^; R^4^; R^5^; R^6^; R^7^	Notum IC_50_ (μM) a
**2a**	–	0.093 ± 0.020
**4a**	2-Me	2.4 ± 1.2
**4b**	3-Me	0.26 ± 0.08
**4c**	3,3-Me_2_	1.4 ± 0.4
**4d**		0.061 ± 0.012
**4e**		1.8 ± 1.2
**4f**		4.1 ± 0.8
**4g**	4-Cl	0.040 ± 0.024
**4h**	5-Cl	0.095 ± 0.014
**4i**	6-Cl	1.4 ± 0.2
**4j**	7-Cl	6.4 ± 0.9
**4k**	4-Me	0.083 ± 0.016
**4l**	4-CN	1.5 ± 0.2
**4m**	4-F	0.093 ± 0.002
**4n**	4-MeO	0.67 ± 0.07
**4o**	4-CF_3_	0.030 ± 0.004
**4p**	4-cPr	0.29 ± 0.14
**4q**	5-Me	0.22 ± 0.02
**4r**	5-CN	>10 b
**4s**	5-F	0.35 ± 0.04
**4t**	5-MeO	0.67 ± 0.08
**4u**	5-CF_3_	0.52 ± 0.05
**4v**	4-CF_3_,5-Cl	0.0013 ± 0.0003
**4w**	4,5-Cl_2_	0.0025 ± 0.0006
**4x**	6,7-Cl_2_	2.2 ± 0.6
**4y**	, 4-Cl	0.0035 ± 0.0006
**4z**	, 5-Cl	0.034 ± 0.010
**4aa**	, 4-CF_3_	0.017 ± 0.002

a All values are mean ± s.d. of n = 4–12 experiments quoted to 2 s.f.

b 20–50% inhibition @ 10 μM.

**Scheme 1:**
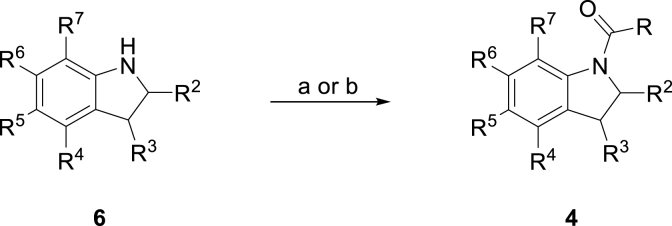
Preparation of Notum inhibitors illustrated with indoline as a representative amine. *Reagents and conditions:* (a) RCOCl (2.0 equiv.), NaHCO_3_ (12 equiv.), CH_2_Cl_2_, 0 °C → RT, 2 h; (b) (i) NaH (1.5 equiv.), THF, 0 °C, (ii) RCOCl (2.0 equiv.), 0 °C → RT, 2 h.

Notum inhibitors **3**–**5** (Table 1, Table 2, Table 3, Table 4) were either purchased or prepared using established synthetic methods from readily available starting materials (Scheme 1). Where necessary, the synthesis of many of these amines (e.g. **6**) have been reported previously and full details of their preparation have been included in the Experimental Section. Notum inhibition activity was determined using the standard Notum-OPTS biochemical assay using IC_50_ values. These values were used to compare relative activities with covalent inhibitors under carefully regulated assay conditions [26]. Confirmed inhibitors were routinely screened in in vitro ADME assays. Selected inhibitors were screened for inhibition of Notum activity in a TCF/LEF Reporter (Luciferase) cell line with exogenous WNT3a and Notum, and soaked into Notum crystals to determine their binding modes and evidence of MoA.

**Table 3:** Notum inhibition and ADME properties for selected *N*-acyl indolines **4**.

**4v** MW 264 *c*logP 2.8	**4w** 230 2.7		**4y** 222 2.4

*Notum Inhibition*			

**Compound**	**4v**	**4w**	**4y**

**Notum** (core, 81–451C330S)			
OPTS, IC_50_ (nM)	1.3 ± 0.3 (n = 4)	2.5 ± 0.6 (n = 11)	3.5 ± 0.6 (n = 7)
TCF-LEF, EC_50_ (nM)	28 ± 20 (n = 3)	22 ± 14 (n = 6)	35 ± 13 (n = 3)

**Notum** (full length)			
OPTS, IC_50_ (nM)	–	4.9 ± 0.6 (n = 3)	11 ± 1.4 (n = 3)

**ADME Properties**^a^			
**LogD**_**7.4**_	–	3.1	–
**PBS solubility**			
(ug/mL)	1.6	0.4	0.2
**MLM/HLM**			
Cl_int_ (μL/min/mg protein)	650/-	1200/140	900/-
**mouse plasma stability**			
t_½_ (h)	–	>4	–
**MDCK-MDR1**			
AB/BA *P*_app_ (x10^−6^ cm/s)	–	35/21	–
ER		0.60	

a See Supplementary Material Tables S1–S5.

**Table 4:** Fine-tuning of structural features to improve metabolic stability.^a^

Compound	Structure b	Notum IC_50_ (μM) c
**5a**		4.4 ± 0.3
**5b**		1.0 ± 0.3
**5c**		0.73 ± 0.04
**5d**		0.031 ± 0.004
**5e**		8.5 ± 2.1
**5f**		0.18 ± 0.02
**5g**		0.49
**5h**		6.1 ± 0.7
**5i**		1.6–10 d
**5j**		1.2 ± 0.4
**5k**		0.49 ± 0.03
**5l**		2.1 ± 0.31
**5m**		0.077 ± 0.010
**5n**		3.6 ± 0.67
**5o**		>10 e

a For microsomal stability data (MLM), see text and Supplementary Material Table S4.

b Compounds containing chiral centres are racemic mixtures.

c All values are mean ± s.d. of n = 4–8 experiments quoted to 2 s.f.

d **5i** demonstrated a binary variable response 1.6 μM (n = 4) and 10 μM (n = 4).

e 20–50% inhibition @ 10 μM.

Building on *N*-acyl indoline **3a**, a range of *N*-acyl scaffolds **3b**-**3o** were sourced and screened (Table 1). However, all of these changes were detrimental for inhibition of Notum activity compared to **3a**. The modest structural change of an indoline **3a** to the corresponding indole **3b** caused a 10-fold decrease despite being very similar in size and likely to occupy a similar position in the pocket (Fig. S2). Binding affinity in this region may be driven by lipophilicity (over π-stacking) with a preference for sp^3^ carbons at C2 and C3. Tetrahydroquinoline **3c** was 6-fold weaker then **3a**, which is due to the larger A-ring (Fig. S2). Overlay of **3c** with **3a** would suggest that the expanded A-ring of **3c** extends forward and may clash with Ala342 at the front of the pocket and so **3c** would be required to sit deeper in the pocket disrupting the interaction with the water bridging to the oxyanion hole.

Loss of activity was observed for a variety of A-ring sizes (5, 6) and *N*-positions (α, β) that resulted in an unfavourable change in the acyl vector (**3a** vs **3c**-**3e**). Deconstructing the [6,5]-ring system into monocyclic (**3f**, **3g**) or acyclic (**3h**) systems resulted in a substantial loss in activity. Oxidation of the A-ring through introduction of a carbonyl group at the 2- or 3- positions was tolerated with a preference for the 3-position (**3i**) showing some polarity could be incorporated into the template. Exchanging the indoline for alternative *N*-acyl benzo heterocycles (**3k**-**3o**) was explored and were inferior to **3a**.

Hence, **3a** was selected as the preferred scaffold for further investigation through sequential substitution at each of the positions on the indoline rings; preferred groups at these positions were then combined to see if the effects were additive to produce inhibitors with greatly improved activity (Table 2). Introduction of small alkyl groups onto the A-ring gave a significant boost in activity. A single 3-Me (**4b**) gave a 2-fold increase but this gain was lost when geminal 3,3-Me_2_ (**4c**) were present. Constraining the two Me into a 3,3-spiro cPr (**4d**: IC_50_ 0.061 μM) gave the most potent inhibitor so far although the corresponding 3,3-spiro cBu (**4e**) or 2,3-fused cPr (**4f**) were less well accommodated, probably due to the increased bulk at the opening to the palmiteolate pocket.

Substitution on the aromatic B-ring was initially explored by walking a Cl substituent around the scaffold (**4g**-**4j**). A 4-Cl (**4g**: IC_50_ 0.040 μM) gave a 10-fold increase in activity over **3a** with a 5-Cl (**4h**: IC_50_ 0.095 μM) also showing an improvement, and these positions were prioritised (4-Cl > 5-Cl > H ≫ 6-Cl > 7-Cl). The X-ray structure of **3a** showed there to be space in the pocket adjacent to the 4- and 5-positions on the indoline to accommodate a substituent, whereas the available space near the 6- and 7-positions was limited. Exploring small substituents as alternatives to the Cl (F, Me, CF_3_, CN, OMe) at 4- and 5-positions (**4k**-**4u**) showed the 4-CF_3_ (**4o**: IC_50_ 0.030 μM) achieved a similar level of activity to 4-Cl but that Cl was favoured in the 5-position. Combining these preferred groups (4-Cl, 4-CF_3_, 5-Cl) as dual substituents gave 4-CF_3_-5-Cl (**4v**: IC_50_ 0.0013 μM) and 4,5-Cl_2_ (**4w**: IC_50_ 0.0025 μM), which achieved still better potency and inhibitors with single digit nanomolar activity. Similar gains in Notum activity were observed when combining a preferred aryl substituent (4-Cl, 4-CF_3_ or 5-Cl) with a 3,3-spiro cPr group (**4y**-**4aa**) with **4y** (IC_50_ 0.0035 μM) as the most potent inhibitor from this subset. In summary, SAR studies in the *N*-acyl indoline template has identified inhibitors (**4v**, **4w**, **4y**) with a similar level of activity to the most potent Notum inhibitors reported [8,9]. Indoline **4w** is still a relatively small molecule (MW 230, HAC 14, *c*logP 2.7) and was a highly efficient inhibitor of Notum (LE 0.85, LLE 5.9) with modest improvements compared to the original lead **3a** (LE 0.75, LLE 5.0).

**Fig. 3:**
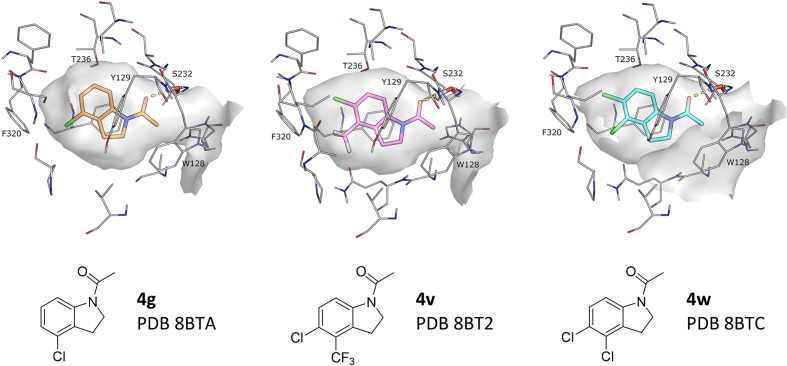
Notum X-ray structures of indolines **4g** (gold), **4v** (pink) and **4w** (sky). For details shown in the figure, see Fig. 2 legend.

In an effort to further understand the binding mode of the indoline series **4**, select compounds were subjected to soaking in Notum crystals and their structures solved (Fig. 3). Notum structures were determined for **4g**, **4v** and **4w** in high resolution and unambiguous ligand electron density.

The structures of **4g**, **4v**, and **4w** were very similar and retained some key features of the binding mode of **3a** such as the water mediated interaction with the oxyanion hole. However, addition of substituents on the aromatic B-ring led to modest shifts in the pocket positioning of these inhibitors compared to **3a**, most likely due to steric interactions with the back of the pocket; the indoline rings move towards Ser232 by ca 1 Å to accommodate the Cl and CF_3_ substituents on the phenyl ring. Overall, these three inhibitors occupy a larger portion of the pocket volume, explaining the increase in Notum activity.

As the programme evolved, integration of the SAR data with available X-ray structures showed that Notum activity for these *N*-acyl indoles **3a** and **4** was largely dependent upon optimum occupancy of the palmitoleate pocket provided that the water mediated H-bond between the ligand carbonyl through to the oxyanion hole was not disrupted.

Early leads **4v**, **4w** and **4y** were then evaluated in additional pharmacology and ADME screens (Table 3). These three compounds restored Wnt signalling in the presence of Notum in a cell-based TCF/LEF (Luciferase) reporter assay and gave regular concentration-response curves up to 10 μM (Fig. S4). Performing these experiments in the absence of Notum showed that the activation of Wnt signalling was due to direct on-target inhibition of Notum and not by assay interference or cell toxicity (up to 10 μM). Screening **4w** and **4y** in the biochemical assay using full length Notum and OPTS confirmed potent inhibition of Notum activity.

Throughout the design cycle, confirmed Notum inhibitors (<100 nM) were routinely screened in in vitro ADME assays to assess their aqueous solubility, microsomal stability and cell permeability (Table 3, Tables S1–S5). It was quickly established that most indolines **4** displayed poor stability in mouse and human liver microsomes (MLM and HLM: t_1/2_ <5 min, Table S4). In contrast, mouse plasma stability of **4w** was excellent with no detectable loss (t_1/2_ >4 h, Table S5).

**Scheme 2:**
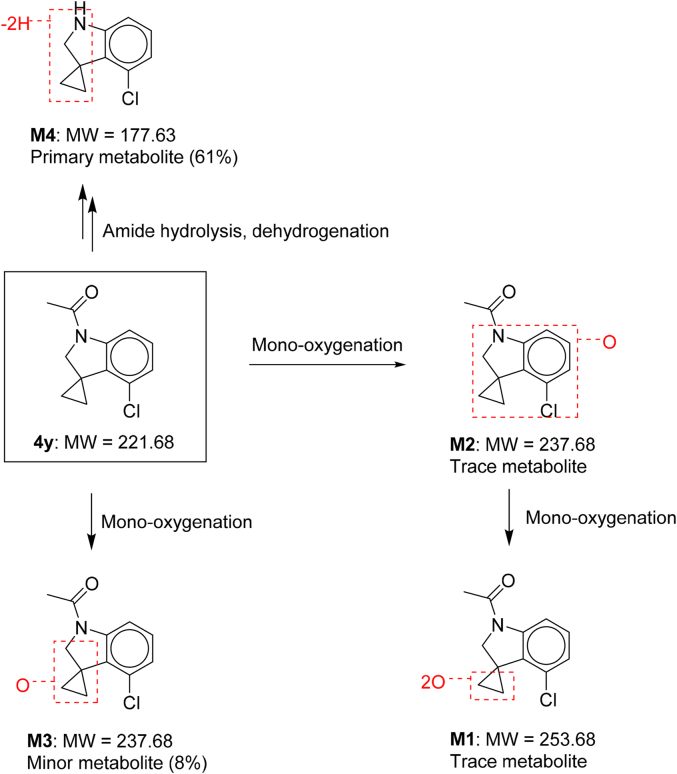
Metabolites and metabolic pathways of **4y** in mouse liver microsomes.

Given the poor microsomal stability of **4**, the main metabolites and routes of metabolism of **4y** were determined in MLM (Scheme 2, Tables S6 and S7). In addition to unchanged parent **4y**, four metabolites of **4y** were detected and identified by LC-UV-MS. These metabolites M1-M4 were assigned: M1: di-oxygenation metabolite; M2-M3: mono-oxygenation metabolites; and M4: amide hydrolysis and dehydrogenation metabolite. Unchanged parent, **4y** accounted for 30% of the total drug-related components in MLM with M4 as the primary metabolite (61%) and M3 as a minor metabolite (8%). M1 and M2 were trace metabolites only detected by MS-MS. The proposed metabolic pathway of **4y** in MLM was amide hydrolysis, dehydrogenation and mono-oxygenation (Scheme 2).

As the *N*-acyl group was a defining motif of the indolines **4** for Notum inhibition (cf **5n**, **5o**), it was likely that microsomal hydrolysis of the amide was a metabolic liability across this series. The next phase of design would need to retain key elements of the pharmacophore for Notum inhibition whilst simultaneously fine-tuning structural features to improve microsomal stability and reduce amide hydrolysis (Table 4).

Increasing the steric bulk of the amide group (**5a**) caused a significant reduction in Notum activity as did replacement of the carboxamide with a sulfonamide (**5b**). Revisiting the N-acyl indole (**5c, 5d**) scaffold with the introduction of preferred aromatic substituents (4-Cl, 4,5-Cl_2_) to enhance Notum inhibition gave an increase in activity over **3b** with the SAR tracking that of the indoline template. However, indole **5d** failed to improve MLM stability. An alternative strategy was to tether the N-acyl group onto the indoline/indole at C2 to create [6,5,5]- and [6,5,6]-tricyclic systems (**5f-5m**). Prototype pyrroloindoline **5f** [27] was encouraging as Notum inhibition was 2-fold superior to the non-cyclised analogue **3a** and MLM stability had improved (**5f**: IC_50_ 0.18 μM, t_1/2_ 18 min, Cl_i_ 74 μL/min/mg protein). Introduction of a Cl group at the preferred aromatic sites (**5j**: 7-Cl and **5k**: 8-Cl) failed to improve Notum activity and caused a modest decrease compared to **5f** indicating a divergence of SAR between these scaffolds. Substitution on the pyrrolidinone ring with geminal 2,2-F_2_ **5m** [28] gave an increase in potency over **5f** albeit with a similar level of MLM stability (**5m**: IC_50_ 0.070 μM, t_1/2_ 16 min, Cl_i_ 91 μL/min/mg protein).

**Fig. 4:**
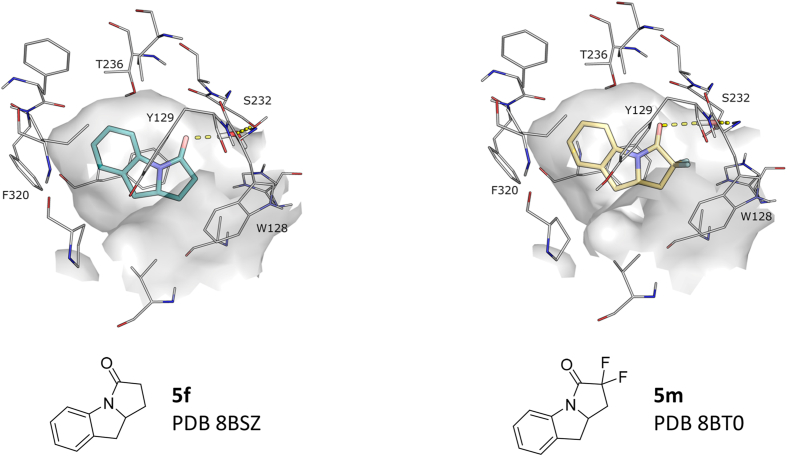
Notum X-ray structures of pyrroloindolines **5f** (teal) and **5m** (yellow). For details shown in the figure, see Fig. 2 legend.

X-ray structures of **5f** and **5m** revealed a shared binding mode very similar to **3a** with key interactions being aromatic stacking to Phe268, Tyr129 and Phe320, and a water bridging the interaction with the oxyanion hole (Fig. 4). It was not immediately clear from the binding mode of these pyrroloindolines why introduction of Cl substituents at preferred aromatic sites fails to improve activity for this template (**5j**, **5k** v **5f**). It could be possible that the shifted binding of the Cl-substituted inhibitors places the framework of the pyrrolidinone ring in a position that would clash with Ala342 at the opening of the pocket (cf 2-Me of **4a**).

From this set of compounds, **4** and **5**, it had not proved possible to combine potent inhibition of Notum activity (IC_50_ < 10 nM) with acceptable microsomal stability (Cl_i_ < 20 μL/min/mg protein), and so further optimisation of this series to identify a brain penetrant tool for in vivo use was not merited as better tools are currently available [29].

## 3 Conclusion

We have described *N*-acyl indolines **4** as potent, non-covalent Notum inhibitors developed from a covalent virtual screening hit **2a**. The lead compounds were simple to synthesise, achieved excellent potency in a biochemical Notum-OPTS assay (IC_50_ < 10 nM) and were shown to restore Wnt signalling in the presence of Notum in a in a cell-based TCF/LEF reporter assay (EC_50_ < 40 nM).

Multiple high resolution X-ray structures established the binding mode of these *N*-acyl indolines. The indoline bound centred in the palmiteolate pocket with aromatic stacking to Phe268, Tyr129, and Phe320 and a water mediated hydrogen bond to the oxyanion hole. Indolines **4v** and **4w** are two potent inhibitors of Notum with SAR and X-ray structures clearly showing that incorporation of 4,5-Cl_2_ and 4-CF_3_-5-Cl substituents were beneficial to binding interactions (cf **3a**).

The *N*-acyl group was an essential motif of the indoline pharmacophore for Notum inhibition although it proved challenging to combine potent inhibition of Notum with acceptable microsomal stability as hydrolysis of the amide bond was the primary metabolic liability in MLM as shown by metabolite identification studies.

We believe these *N*-acyl indolines **4** will be useful tools for use in *in vitro* studies to investigate the role of Notum in disease models, especially when paired with a structurally related covalent inhibitor (e.g. **4w** and **2a**). Overall, this study highlights the designed switch from covalent to non-covalent Notum inhibitors and so illustrates a complementary approach for hit generation and target inhibition.

## 4 Experimental Section

### 4.1 Notum assay materials and methods

#### 4.1.1 Notum protein expression and purification

Methods have been described in detail [21,22].

#### 4.1.2 Notum OPTS biochemical assay

Methods have been described in detail [21,22].

#### 4.1.3 TCF/LEF reporter (Luciferase) cell based assay

Methods have been described in detail [21,22].

### 4.2 Structural biology

The methods of protein production, crystallization, data collection and structure determination have been described in detail [21,22]. X-ray structure determination for **3a** (8BT8), **4g** (8BTA), **4v** (8BT2), **4w** (8BTC), **5f** (8BSZ), and **5m** (8BT0) have been deposited and released in the Protein Data Bank (PDB). Electron density omit maps are presented in Fig. S1.

To prepare the figures of the crystal structures the PDB entry was processed using the Protein Preparation Wizard in Schrödinger Maestro 12.4 using the default settings and optimizing the hydrogen bond assignment. No changes were made to the position of heavy atoms. Final images were prepared using PyMol version 2.4.

### 4.3 ADME protocols and results

Molecular properties (MW, *c*logP) were calculated with ChemDraw Professional v16.0.1.4(77). Distribution coefficients (LogD_7.4_) were measured using the shake flask method.

Selected compounds were routinely screened for aqueous solubility in PBS (pH 7.4), transit performance in MDCK-MDR1 cell lines for permeability, and metabolic stability in MLM and HLM as a measure of clearance [21,29]. The main metabolites and routes of metabolism of **4y** were determined in MLM. Assay protocols and additional data are presented in the Supplementary Material (Tables S1–S7). ADME studies reported in this work were performed by WuXi AppTec (Shanghai, China).

### 4.4 Chemistry

#### 4.4.1 General information

Unless preparative details are provided, all reagents were purchased from commercial suppliers and used without further purification. Solvents were of ACS reagent grade or higher and purchased from commercial suppliers without further purification. Microwave assisted reactions were performed in a Biotage Initiator+. Thin-layer chromatography (TLC) was carried out on aluminium-backed silica plates. The plates were visualized under UV (254 nm) light, followed by staining with phosphomolybdic acid dip or potassium permanganate and gentle heating. Organic solvent layers were routinely dried with anhydrous Na_2_SO_4_ or MgSO_4_ and concentrated using a Büchi rotary evaporator. Compound purification by column chromatography was performed using a Biotage Isolera using prepacked Biotage SNAP KP-Sil silica cartridges or Biotage SNAP Ultra C18 reverse phase cartridges. ^1^H and ^13^C NMR spectra were recorded in deuterated (≥99.5%) solvents on either a Bruker Avance 400 (400 MHz), Bruker Avance 600 (600 MHz), or Bruker Avance 700 (700 MHz). Chemical shifts (*δ*) are reported as parts per million (ppm), coupling constants (J) are reported in Hz, and signal multiplicities are reported as singlet (s), doublet (d), triplet (t), quartet (q), pentet (p), sextet (sext), doublet of doublets (dd), doublet of triplets (dt), triplet of doublets (td), triplet of triplets (tt), multiplet (m), broad (br) or apparent (app). Liquid chromatography−mass spectrometry (LCMS) analysis was performed on a Waters Acquity H-Class UPLC system with either an acidic (HSS C18 Column, H_2_O/MeCN, 0.1% formic acid) or a basic (BEH C18 Column, H_2_O/MeCN, 10 mM NH_4_OH) mobile phase. The observed mass and isotope pattern matched the corresponding theoretical values as calculated from the expected elemental formula.

Most yields for chemical reactions are for single experiments and have not been optimised. The purity of compounds **3–5** was evaluated by NMR spectroscopy and LCMS analysis. All compounds had purity ≥95% unless otherwise stated.

Notum inhibitors **3**–**5** were either purchased or prepared using established synthetic methods from readily available starting materials. Chemical Abstracts Service (CAS) Registry Numbers are provided for these known compounds: **3a**, 16078-30-1; **3b**, 576-15-8; **3c**, 4168-19-1; **3d**, 18913-38-1; **3e**, 14028-76-2; **3f**, 55692-54-1; **3g**, 4641-57-0; **3h**, 573-26-2; **3i**, 16800-86-3; **3j**, 21905-78-2; **3k**, 13436-49-2; **3l**, 18773-95-0; **3m**, 70801-52-4; **3n**, 6397-16-6; **3o**, 79602-52-1; **4a**, 131880-76-7; **4b**, 31676-46-7; **4c**, 16078-32-3; **4d**, 944828-13-1; **4e**, 2124766-25-0; **4f**, 2250235-49-3; **4g**, 860024-86-8; **4h**, 25630-01-7; **4i**, 68748-67-4; **4j**, 114144-23-9; **4k**, 165250-67-9; **4l**, 1537122; **4m**, 860024-83-5; **4n**, 91132-99-2; **4o**, 2248828-38-6; **4q**, 950589-59-0; **4r**, 15861-29-7; **4s**, 2354-90-7; **4t**, 4770-33-6; **4u**, 1440538-41-9; **4z**, 2250235-57-3; **5c**, 1261240-69-0; **5e**, 1444593-72-9; **5f**, 65481-10-9; **5g**, 26709-65-9; **5h**, 1824329-88-5; **5j**, 2058245-08-0; **5l**, 1282060-48-3; **5m**, 2222800-57-7; **5n**, 162100-51-8; **5o**, 103628-32-6.

#### 4.4.2 General Methods

##### 4.4.2.1 Preparation of *N*-acyl amides

*General Method 1.1*. NaHCO_3_ (460 mg, 5.40 mmol, 12.0 equiv.) was added to the amine (0.45 mmol, 1.0 equiv.) in CH_2_Cl_2_ (3.0 mL). The reaction mixture was cooled to 0 °C (external), then acetyl chloride (60 μL, 0.90 mmol, 2.0 equiv.) was added dropwise. The ice bath was removed, and the mixture was stirred at RT for 2 h, H_2_O (20 mL) and CH_2_Cl_2_ (20 mL) were cautiously added, and then vigorously stirred for 5 min. The organic phase was dried, filtered, and concentrated under reduced pressure. Amides isolated with <95% purity (determined by LCMS and ^1^H NMR) were further purified by reverse phase chromatography.

*General Method 1.2*. Sodium hydride (32 mg, 1.4 mmol, 1.5 equiv.) was added portionwise to the amine (0.9 mmol, 1.0 equiv.) in THF (6.0 mL) at 0 °C (external). Once gas evolution had ceased, acetyl chloride (130 μL, 1.8 mmol, 2.0 equiv.) was added cautiously, the ice bath was removed and the mixture was stirred at RT for 2 h H_2_O (20 mL) and CH_2_Cl_2_ (20 mL) were then cautiously added, and vigorously stirred for 5 min. The organic phase was dried, filtered, and concentrated under reduced pressure. The crude material was purified by reverse phase chromatography to give the amide.

#### 4.4.3 Notum inhibitors

##### 4.4.3.1 1-(Indolin-1-yl)ethan-1-one (**3a**)

Purchased from Sigma Aldrich, 379492-5G.

##### 4.4.3.2 1-(1*H*-Indol-1-yl)ethan-1-one (**3b**)

Purchased from Sigma Aldrich, 377104-1G.

##### 4.4.3.3 1-(3,4-Dihydroquinolin-1(2*H*)-yl)ethan-1-one (**3c**)

Prepared by General Method 1.1 from 1,2,3,4-tetrahydroquinoline. Isolated as a brown oil (326 mg, 37%).

^1^H NMR (700 MHz, CDCl_3_) *δ* 7.20–7.07 (m, 4H), 3.79 (s, 2H), 2.72 (t, *J* = 5.7 Hz, 2H), 2.23 (s, 3H), 1.96 (app p, *J* = 6.6 Hz, 2H); ^13^C NMR (176 MHz, CDCl_3_) *δ* 170.3, 139.4, 133.7, 128.5, 126.2, 125.3, 124.7, 42.8, 27.0, 24.2, 23.2; LCMS *m/z* 176.1 [M+H]^+^.

##### 4.4.3.4 1-(Isoindolin-2-yl)ethan-1-one (**3d**)

Prepared by General Method 1.1 from isoindoline. Isolated as an off-white solid (24 mg, 3%).

^1^H NMR (700 MHz, DMSO‑*d*_6_) *δ* 7.37–7.27 (m, 4H), 4.82 (s, 2H), 4.61 (s, 2H), 2.06 (s, 3H); ^13^C NMR (176 MHz, DMSO‑*d*_6_) *δ* 168.6, 137.0, 136.4, 127.4, 127.3, 123.0, 122.8, 52.5, 51.4, 22.0; LCMS *m/z* 162.1 [M+H]^+^.

##### 4.4.3.5 1-(3,4-Dihydroisoquinolin-2(1*H*)-yl)ethan-1-one (**3e**)

Prepared by General Method 1.1 from 1,2,3,4-tetrahydroisoquinoline. Isolated as a pale yellow oil (439 mg, 50%). NMR spectra show rotamers (6:4) consistent with previous reports [30].

^1^H NMR (700 MHz, DMSO‑*d*_6_) *δ* 7.20–7.15 (m, 4H), 4.63 (s, 0.8 H), 4.58 (s, 1.2H), 3.64 (t, *J* = 6.0 Hz, 2H), 2.85 (t, *J* = 6.0 Hz, 1.2H), 2.77–2.72 (m, 0.8H), 2.08 (s, 1.2 H), 2.07 (s, 1.8H); ^13^C NMR (176 MHz, DMSO‑*d*_6_) *δ* 168.7, 168.6, 134.8, 134.6, 133.7, 133.4, 128.5, 128.4, 126.4, 126.4, 126.3, 126.3, 126.1, 126.1, 47.1, 43.32, 43.31, 38.8, 28.7, 28.0, 21.8, 21.4; LCMS *m/z* 176.1 [M+H]^+^.

##### 4.4.3.6 1-(3-Phenylpyrrolidin-1-yl)ethan-1-one (**3f**)

Prepared by General Method 1.1 from 2-phenylpyrrolidine. Isolated as a clear oil (62 mg, 7%).

^1^H NMR (700 MHz, CDCl_3_) *δ* 7.37–7.28 (m, 3H), 7.15 (d, *J* = 7.6 Hz, 2H), 4.96–4.91 (m, 1H), 3.81–3.70 (m, 3H), 2.46–2.38 (m, 1H), 2.06–1.96 (m, 2H), 1.91 (s, 3H); ^13^C NMR (176 MHz, CDCl_3_) *δ* 171.1, 142.8, 129.1, 127.6, 125.5, 62.7, 47.5, 36.4, 22.3, 22.1; LCMS *m/z* 190.2 [M+H]^+^.

##### 4.4.3.7 1-Phenylpyrrolidin-2-one (**3g**)

Purchased from Key Organics, FS-2711.

##### 4.4.3.8 *N*-Methyl-*N*-(*o*-tolyl)acetamide (**3h**)

Prepared by General Method 1.1 from *N*,2-dimethylaniline. Isolated as a pale yellow oil (63 mg, 8%).

^1^H NMR (700 MHz, DMSO‑*d*_6_) *δ* 7.73–7.41 (m, 4H), 3.38 (s, 3H), 2.52 (s, 3H), 1.96 (s, 3H); ^13^C NMR (176 MHz, DMSO‑*d*_6_) *δ* 169.0, 142.9, 135.1, 131.2, 128.2, 128.0, 127.5, 35.3, 21.6, 16.9; LCMS *m/z* 164.1 [M+H]^+^.

##### 4.4.3.9 1-Acetylindolin-3-one (**3i**)

Purchased from Activate Scientific, AS39248.

##### 4.4.3.10 1-Acetylindolin-2-one (**3j**)

Prepared by General Method 1.2 from 2-oxindole. Isolated as an off-white solid (67 mg, 43%).

^1^H NMR (600 MHz, DMSO‑*d*_6_) *δ* 8.08 (d, *J* = 8.1 Hz, 1H), 7.37–7.28 (m, 2H), 7.20–7.16 (m, 1H), 3.82 (s, 2H), 2.55 (s, 3H); ^13^C NMR (151 MHz, DMSO‑*d*_6_) *δ* 175.4, 170.7, 141.2, 127.5, 124.8, 124.6, 124.3, 115.6, 36.3, 26.4; LCMS *m/z* 176.1 [M+H]^+^.

##### 4.4.3.11 1-(1*H*-Indazol-1-yl)ethan-1-one (**3k**)

Purchased from Key Organics, DS-6890.

##### 4.4.3.12 1-(1*H*-Benzo[*d*]imidazole-1-yl)ethan-1-one (**3l**)

Benzimidazole (0.20 g, 1.7 mmol), Ac_2_O (3.5 mL, 37.0 mmol) and AcOH (5.0 mL, 87.3 mmol) were stirred at RT for 0.5 h. The reaction mixture was concentrated under reduced pressure, and then azeotroped with PhMe (x3) to give 1-(1*H*-benzo[*d*]imidazole-1-yl)ethan-1-one (270 mg, quant.) as an off-white solid.

^1^H NMR (600 MHz, DMSO‑*d*_6_) *δ* 8.89 (s, 1H), 8.17 (d, *J* = 7.5 Hz, 1H), 7.76 (d, *J* = 7.9 Hz, 1H), 7.44–7.35 (m, 2H), 2.75 (s, 3H); ^13^C NMR (151 MHz, DMSO‑*d*_6_) *δ* 169.1, 144.2, 143.7, 131.2, 125.3, 124.6, 120.0, 115.2, 23.7; LCMS *m/z* 161.0 [M+H]^+^.

##### 4.4.3.13 1-(2,3-Dihydro-4*H*-benzo[*b*][1,4]oxazin-4-yl)ethan-1-one (**3m**)

Prepared by General Method 1.1 from 3,4-dihydro-2*H*-1,4-benzoxazine. Isolated as an off-white solid (35 mg, 43%).

^1^H NMR (700 MHz, methanol-*d*_*4*_) *δ* 8.31–6.64 (m, 4H), 4.36–4.22 (m, 2H), 3.90 (s, 2H), 2.29 (s, 3H); ^13^C NMR (176 MHz, methanol-*d*_*4*_) *δ* 171.5, 148.4, 126.7, 125.4, 122.4, 121.0, 118.2, 67.9, 40.9, 22.9; LCMS *m/z* 178.1 [M+H]^+^.

##### 4.4.3.14 1-(2,3-Dihydro-4*H*-benzo[*b*][1,4]thiazin-4-yl)ethan-1-one (**3n**)

Prepared by General Method 1.1 from 3,4-dihydro-2*H*-1,4-benzothiazine. Isolated as an off-white solid (85 mg, 98%).

^1^H NMR (600 MHz, DMSO‑*d*_6_) *δ* 7.37 (d, *J* = 6.9 Hz, 1H), 7.31–7.02 (m, 3H), 3.84 (s, 2H), 3.28–3.08 (m, 2H), 2.09 (s, 3H); ^13^C NMR (151 MHz, DMSO‑*d*_6_) *δ* 159.1, 128.4, 118.7, 117.10, 117.06, 116.7, 114.8, 30.8, 18.4, 12.7; LCMS *m/z* 194.1 [M+H]^+^.

##### 4.4.3.15 1-(1,1-Dioxido-2,3-dihydro-4*H*-benzo[*b*][1,4]thiazin-4-yl)ethan-1-one (**3o**)

NaHCO_3_ (460 mg, 5.40 mmol, 12.0 equiv.) was added to a solution of 3,4-dihydro-2*H*-1,4-benzothiazine (68 mg, 0.45 mmol, 1.0 equiv.) in CH_2_Cl_2_ (3.0 mL). The mixture was cooled to 0 °C (external), and then acetyl chloride (60 μL, 0.90 mmol, 2.0 equiv.) was added dropwise. The ice bath was removed, and the reaction mixture was stirred at RT for 2 h H_2_O (10 mL) was then cautiously added and the mixture vigorously stirred for 5 min. The organic phase containing **3n** was separated, cooled to 0 °C (external), 3-chloroperbenzoic acid (250 mg, 1.1 mmol, 2.5 equiv.) was added portionwise, and the reaction mixture allowed to warm slowly to RT over 16 h. The mixture was diluted with sat. aq. NaHCO_3_ (20 mL) and extracted with CH_2_Cl_2_ (20 mL). The organic phase was dried, filtered, and concentrated under reduced pressure. The crude product was purified by reverse phase chromatography (0.1% formic acid modifier). Isolated as a colourless wax (65 mg, 64%).

^1^H NMR (700 MHz, CDCl_3_) *δ* 7.95 (dd, *J* = 7.9, 1.4 Hz, 1H), 7.62–7.54 (m, 1H), 7.45 (t, *J* = 7.7 Hz, 1H), 7.33 (d, *J* = 8.0 Hz, 1H), 4.45–4.39 (m, 2H), 3.63–3.55 (m, 2H), 2.25 (s, 3H); ^13^C NMR (176 MHz, CDCl_3_) *δ* 169.8, 138.3, 133.2, 133.2, 127.4, 125.7, 125.1, 54.3, 42.4, 22.9; LCMS *m/z* 226.0[M+H]^+^.

##### 4.4.3.16 1-(2-Methylindolin-1-yl)ethan-1-one (**4a**)

Prepared by General Method 1.1 from 2-methylindoline. Isolated as an off-white solid (703 mg, 80%).

^1^H NMR (600 MHz, DMSO‑*d*_6_) *δ* 8.03–7.96 (m, 1H), 7.33–7.08 (m, 2H), 7.00 (t, *J* = 7.5 Hz, 1H), 4.80–4.47 (m, 1H), 3.42–3.18 (m, 1H), 2.71–2.57 (m, 1H), 2.38–2.14 (m, 3H), 1.34–1.00 (m, 3H); ^13^C NMR (176 MHz, DMSO‑*d*_6_) *δ* 160.0, 141.5, 130.8, 127.0, 125.2, 123.4, 116.9, 55.6, 35.8, 23.2, 21.4; LCMS *m/z* 176.1 [M+H]^+^.

##### 4.4.3.17 1-(3-Methylindolin-1-yl)ethan-1-one (**4b**)

Prepared by General Method 1.1 from 3-methyl-2,3-dihydro-1*H*-indole hydrochloride. Isolated as an off-white solid (65 mg, 63%).

^1^H NMR (600 MHz, DMSO‑*d*_6_) *δ* 8.01 (d, *J* = 8.0 Hz, 1H), 7.24 (d, *J* = 7.4 Hz, 1H), 7.14 (t, *J* = 7.7 Hz, 1H), 7.00 (td, *J* = 7.4, 0.9 Hz, 1H), 4.26 (t, *J* = 9.9 Hz, 1H), 3.61 (dd, *J* = 10.3, 6.9 Hz, 1H), 3.52–3.39 (m, 1H), 2.15 (s, 3H), 1.28 (d, *J* = 6.9 Hz, 3H); ^13^C NMR (176 MHz, DMSO‑*d*_6_) *δ* 168.5, 142.4, 136.8, 127.1, 123.7, 123.2, 115.7, 56.0, 34.0, 24.0, 20.0; LCMS *m/z* 176.1 [M+H]^+^.

##### 4.4.3.18 1-(3,3-Dimethylindolin-1-yl)ethan-1-one (**4c**)

Prepared by General Method 1.1 from 3,3-dimethylindoline hydrochloride. Isolated as an off-white solid.

^1^H NMR (700 MHz, DMSO‑*d*_6_) *δ* 8.01 (d, *J* = 8.0 Hz, 1H), 7.23 (d, *J* = 7.2 Hz, 1H), 7.18–7.11 (m, 1H), 7.01 (t, *J* = 7.1 Hz, 1H), 3.84 (s, 2H), 2.15 (s, 3H), 1.30 (s, 6H); ^13^C NMR (176 MHz, DMSO‑*d*_6_) *δ* 168.6, 141.5, 140.8, 127.2, 123.3, 122.2, 115.7, 62.5, 39.8, 28.4, 24.0; LCMS *m/z* 190.1 [M+H]^+^.

##### 4.4.3.19 1-(Spiro[cyclopropane-1,3′-indolin]-1′-yl)ethan-1-one (**4d**)

Prepared by General Method 1.1 from 1′,2′-dihydrospiro[cyclopropane-1,3′-indole] hydrochloride. Isolated as an off-white solid (78 mg, 93%).

^1^H NMR (700 MHz, DMSO‑*d*_6_) *δ* 8.02 (d, *J* = 8.0 Hz, 1H), 7.11–7.07 (m, 1H), 6.94 (td, *J* = 7.4, 0.9 Hz, 1H), 6.78 (dd, *J* = 7.4, 0.6 Hz, 1H), 4.09 (s, 2H), 2.12 (s, 3H), 1.11–1.08 (m, 2H), 1.04–1.01 (m, 2H); ^13^C NMR (176 MHz, DMSO‑*d*_6_) *δ* 168.5, 143.2, 136.8, 126.4, 123.3, 118.6, 115.3, 56.1, 23.9, 22.3, 17.0; LCMS *m/z* 188.1 [M+H]^+^.

##### 4.4.3.20 1-(Spiro[cyclobutane-1,3′-indolin]-1′-yl)ethan-1-one (**4e**)

Prepared by General Method 1.1 from spiro[cyclobutane-1,3′-indoline] hydrochloride. Isolated as an off-white solid (85 mg, 83%). NMR spectra show rotamers.

^1^H NMR (600 MHz, CDCl_3_) *δ* 8.16 (d, *J* = 8.1 Hz, 1H), 7.49–7.40 (m, 1H), 7.21 (dd, *J* = 11.2, 4.4 Hz, 1H), 7.10 (t, *J* = 7.5 Hz, 1H), 4.22–4.08 (m, 2H), 2.46–2.35 and 2.30–2.21 (2 x m, 7H), 2.12–2.00 (m, 2H); ^13^C NMR (151 MHz, CDCl_3_) *δ* 168.6, 141.9, 139.1, 128.1, 124.1, 122.3, 116.7, 63.6, 46.3, 36.5, 24.4, 16.1; LCMS *m/z* 202.1 [M+H]^+^.

##### 4.4.3.21 1-(6b-Methyl-1a,6b-dihydrocyclopropa[*b*]indol-2(1*H*)-yl)ethan-1-one (**4f**) [31]

*N*-(2-Bromophenyl)-*N*-(2-methylallyl)acetamide (80 mg, 0.3 mmol, 1.0 equiv), Pd(PPh_3_)_4_ (35 mg, 0.03 mmol, 0.1 equiv.), K_2_CO_3_ (50 mg, 0.36 mmol, 1.2 equiv.), pivalic acid (31 mg, 0.3 mmol, 1.0 equiv), 4 Å molecular sieve (40 mg), then DMSO (1.0 mL) were added to an oven-dried flask under argon, and then heated to 125 °C for 16 h. The reaction was cooled to RT, diluted with sat. aq. NH_4_Cl (2.0 mL), and extracted with EtOAc (2 × 10 mL). The organic phase was dried, filtered, and concentrated under reduced pressure. The residue was purified by column chromatography using 25% EtOAc in cyclohexane as eluant. Isolated as an off-white solid (25 mg, 45%)

^1^H NMR (600 MHz, CDCl_3_) *δ* 8.14 (d, *J* = 8.1 Hz, 1H), 7.28 (d, *J* = 7.4 Hz, 1H), 7.18 (t, *J* = 7.8 Hz, 1H), 7.05 (t, *J* = 7.4 Hz, 1H), 3.70 (dd, *J* = 5.9, 2.5 Hz, 1H), 2.37 (s, *J* = 7.0 Hz, 3H), 1.58 (s, 3H), 1.07 (t, *J* = 5.7 Hz, 1H), 0.53 (dd, *J* = 5.4, 2.5 Hz, 1H); ^13^C NMR (151 MHz, CDCl_3_) *δ* 169.2, 141.6, 136.7, 127.2, 123.4, 122.6, 117.5, 45.5, 26.1, 24.6, 18.6, 17.8; LCMS *m/z* 188.1 [M+H]^+^.

##### 4.4.3.22 1-(4-Chloroindolin-1-yl)ethan-1-one (**4g**)

Prepared by General Method 1.1 from 4-chloro-2,3-dihydro-1*H*-indole hydrochloride. Isolated as an off-white solid (233 mg, 91%).

^1^H NMR (600 MHz, DMSO‑*d*_6_) *δ* 7.98 (d, *J* = 8.0 Hz, 1H), 7.18 (t, *J* = 8.0 Hz, 1H), 7.04 (d, *J* = 8.0 Hz, 1H), 4.14 (t, *J* = 8.6 Hz, 2H), 3.14 (t, *J* = 8.6 Hz, 2H), 2.15 (s, 3H); ^13^C NMR (176 MHz, DMSO‑*d*_6_) *δ* 335.7, 311.2, 296.7, 296.1, 295.9, 289.4, 281.0, 214.7, 193.6, 190.7; LCMS *m/z* 196.0 [M+H]^+^.

##### 4.4.3.23 1-(5-Chloroindolin-1-yl)ethan-1-one (**4h**)

Prepared by General Method 1.1 from 5-chloro-2,3-dihydro-1*H*-indole hydrochloride. Isolated as an off-white solid (336 mg, 34%).

^1^H NMR (700 MHz, DMSO‑*d*_6_) *δ* 8.01 (d, *J* = 8.6 Hz, 1H), 7.28 (s, 1H), 7.18 (d, *J* = 8.6 Hz, 1H), 4.10 (t, *J* = 8.6 Hz, 2H), 3.14 (t, *J* = 8.5 Hz, 2H), 2.15 (s, 3H); ^13^C NMR (176 MHz, DMSO‑*d*_6_) *δ* 168.7, 141.9, 134.4, 126.7, 126.5, 124.7, 116.7, 48.3, 27.2, 23.8; LCMS *m/z* 196.1 [M+H]^+^.

##### 4.4.3.24 1-(6-Chloroindolin-1-yl)ethan-1-one (**4i**)

Prepared by General Method 1.1 from 6-chloro-2,3-dihydro-1*H*-indole hydrochloride. Isolated as an off-white solid (93 mg, 90%).

^1^H NMR (600 MHz, DMSO‑*d*_6_) *δ* 8.04 (d, *J* = 2.0 Hz, 1H), 7.23 (d, *J* = 7.9 Hz, 1H), 7.02 (dd, *J* = 7.9, 2.1 Hz, 1H), 4.12 (t, *J* = 8.6 Hz, 2H), 3.11 (t, *J* = 8.5 Hz, 2H), 2.16 (s, 3H); ^13^C NMR (176 MHz, DMSO‑*d*_6_) *δ* 169.1, 144.2, 131.1, 131.0, 126.0, 122.6, 115.4, 48.7, 26.9, 23.9; LCMS *m/z* 196.0 [M+H]^+^.

##### 4.4.3.25 1-(7-Chloroindolin-1-yl)ethan-1-one (**4j**)

Prepared by General Method 1.1 from 7-chloro-2,3-dihydro-1*H*-indole hydrochloride. Isolated as an off-white solid (77 mg, 75%).

^1^H NMR (600 MHz, DMSO‑*d*_6_) *δ* 7.25 (dd, *J* = 7.3, 1.1 Hz, 1H), 7.22 (dd, *J* = 8.1, 0.8 Hz, 1H), 7.10–7.06 (m, 1H), 4.09 (t, *J* = 7.6 Hz, 2H), 3.05 (t, *J* = 7.6 Hz, 2H), 2.19 (s, 3H); ^13^C NMR (176 MHz, DMSO‑*d*_6_) *δ* 168.5, 140.3, 138.2, 128.2, 126.0, 123.3, 123.3, 51.0, 29.7, 23.4; LCMS *m/z* 196.0 [M+H]^+^.

##### 4.4.3.26 1-(4-Methylindolin-1-yl)ethan-1-one (**4k**)

Prepared by General Method 1.1 from 4-methyl-2,3-dihydro-1*H*-indole hydrochloride. Isolated as an off-white solid (72 mg, 91%).

^1^H NMR (600 MHz, DMSO‑*d*_6_) *δ* 7.86 (d, *J* = 8.0 Hz, 1H), 7.03 (t, *J* = 7.8 Hz, 1H), 6.80 (d, *J* = 7.5 Hz, 1H), 4.10–4.06 (m, 2H), 3.03 (t, *J* = 8.5 Hz, 2H), 2.18 (s, 3H), 2.14 (s, 3H); ^13^C NMR (151 MHz, DMSO‑*d*_6_) *δ* 168.5, 142.6, 133.7, 130.4, 127.1, 124.0, 113.3, 48.1, 26.2, 24.1, 18.3; LCMS *m/z* 176.1 [M+H]^+^.

##### 4.4.3.27 1-Acetylindoline-4-carbonitrile (**4l**)

Prepared by General Method 1.1 from 2,3-dihydro-1*H*-indole-4-carbonitrile. Isolated as an off-white solid (77 mg, 92%).

^1^H NMR (600 MHz, CDCl_3_) *δ* 8.41 (dt, *J* = 10.1, 5.1 Hz, 1H), 7.31–7.25 (m, 2H), 4.16 (t, *J* = 8.6 Hz, 2H), 3.39 (t, *J* = 8.6 Hz, 2H), 2.25 (s, 3H); ^13^C NMR (151 MHz, CDCl_3_) *δ* 169.3, 144.0, 135.7, 128.9, 126.4, 121.1, 117.3, 108.9, 48.7, 27.8, 24.3; LCMS *m/z* 187.1 [M+H]^+^.

##### 4.4.3.28 1-(4-Fluoroindolin-1-yl)ethan-1-one (**4m**)

Prepared by General Method 1.1 from 4-fluoro-2,3-dihydro-1*H*-indole hydrochloride. Isolated as an off-white solid (74 mg, 92%).

^1^H NMR (600 MHz, DMSO‑*d*_6_) *δ* 7.85 (d, *J* = 8.0 Hz, 1H), 7.19 (dd, *J* = 14.3, 8.1 Hz, 1H), 6.82 (t, *J* = 8.6 Hz, 1H), 4.15 (t, *J* = 8.6 Hz, 2H), 3.15 (t, *J* = 8.5 Hz, 2H), 2.16 (s, 3H); ^13^C NMR (151 MHz, DMSO‑*d*_6_) *δ* 168.8, 158.5 (d, *J* = 242.1 Hz), 145.5 (d, *J* = 8.6 Hz), 129.4 (d, *J* = 7.9 Hz), 117.7 (d, *J* = 22.1 Hz), 112.0 (d, *J* = 3.0 Hz), 109.8 (d, *J* = 19.9 Hz), 48.7, 24.1, 23.7; LCMS *m/z* 180.1 [M+H]^+^.

##### 4.4.3.29 1-(4-Methoxyindolin-1-yl)ethan-1-one (**4n**)

Prepared by General Method 1.1 from 4-methoxyindoline hydrochloride. Isolated as an off-white solid (72 mg, 84%).

^1^H NMR (600 MHz, DMSO‑*d*_6_) *δ* 7.66 (d, *J* = 8.0 Hz, 1H), 7.12 (t, *J* = 8.1 Hz, 1H), 6.65 (d, *J* = 8.2 Hz, 1H), 4.08 (t, *J* = 8.6 Hz, 2H), 3.78 (s, 3H), 2.99 (t, *J* = 8.6 Hz, 2H), 2.13 (s, 3H); ^13^C NMR (151 MHz, DMSO‑*d*_6_) *δ* 168.5, 155.5, 144.2, 128.6, 118.2, 109.0, 106.0, 55.2, 48.5, 24.6, 24.1; LCMS *m/z* 193.1 [M+H]^+^.

##### 4.4.3.30 1-(4-(Trifluoromethyl)indolin-1-yl)ethan-1-one (**4o**)

Prepared by General Method 1.1 from 4-(trifluoromethyl)indoline. Isolated as an off-white solid (87 mg, 84%).

^1^H NMR (700 MHz, DMSO‑*d*_6_) *δ* 8.31 (d, *J* = 8.1 Hz, 1H), 7.38 (t, *J* = 7.9 Hz, 1H), 7.29 (d, *J* = 7.8 Hz, 1H), 4.17 (t, *J* = 8.6 Hz, 3H), 3.28 (t, *J* = 8.4 Hz, 2H), 2.18 (s, 3H); ^13^C NMR (176 MHz, DMSO‑*d*_6_) *δ* 169.1, 144.4, 129.9, 128.3, 125.3 (q, *J* = 31.4 Hz), 124.1 (q, *J* = 272.9 Hz), 119.3, 119.2 (q, *J* = 4.5 Hz), 48.2, 26.4, 24.1; LCMS *m/z* 230.1 [M+H]^+^.

##### 4.4.3.31 1-(4-Cyclopropylindolin-1-yl)ethan-1-one (**4p**)

Prepared by General Method 1.1 from 4-cyclopropyl-2,3-dihydro-1*H*-indole hydrochloride. Isolated as an off-white solid (79 mg, 87%).

^1^H NMR (700 MHz, DMSO‑*d*_6_) *δ* 7.84 (d, *J* = 7.9 Hz, 1H), 7.03 (t, *J* = 7.8 Hz, 1H), 6.51 (d, *J* = 7.7 Hz, 1H), 4.14–4.07 (m, 2H), 3.18 (t, *J* = 8.5 Hz, 2H), 2.14 (s, 3H), 1.86–1.75 (m, 1H), 0.95–0.87 (m, 2H), 0.69–0.61 (m, 2H); ^13^C NMR (176 MHz, DMSO‑*d*_6_) *δ* 168.4, 142.4, 139.3, 130.6, 127.3, 118.1, 113.0, 48.1, 26.2, 24.0, 12.2, 7.7; LCMS *m/z* 202.2 [M+H]^+^.

##### 4.4.3.32 1-(5-Methylindolin-1-yl)ethan-1-one (**4q**)

Prepared by General Method 1.1 from 5-methyl-2,3-dihydro-1*H*-indole. Isolated as an off-white solid (71 mg, 90%).

^1^H NMR (600 MHz, DMSO‑*d*_6_) *δ* 7.90 (d, *J* = 8.1 Hz, 1H), 7.02 (s, 1H), 6.93 (d, *J* = 8.1 Hz, 1H), 4.07–4.02 (m, 2H), 3.08 (t, *J* = 8.5 Hz, 2H), 2.23 (s, 3H), 2.12 (s, 3H); ^13^C NMR (151 MHz, DMSO‑*d*_6_) *δ* 168., 140.7, 132.0, 131.9, 127.3, 125.4, 115.5, 48.2, 27.3, 24.0, 20.6; LCMS *m/z* 176.1 [M+H]^+^.

##### 4.4.3.33 1-Acetylindoline-5-carbonitrile (**4r**)

Prepared by General Method 1.1 from 2,3-dihydro-1H-indole-5-carbonitrile. Isolated as an off-white solid (74 mg, 88%).

^1^H NMR (600 MHz, DMSO‑*d*_6_) *δ* 8.12 (d, *J* = 8.4 Hz, 1H), 7.67 (s, 1H), 7.63 (d, *J* = 8.4 Hz, 1H), 4.15 (t, *J* = 8.7 Hz, 2H), 3.17 (t, *J* = 8.6 Hz, 2H), 2.19 (s, 3H); ^13^C NMR (151 MHz, DMSO‑*d*_6_) *δ* 169.8, 146.8, 133.5, 132.5, 128.4, 119.4, 115.9, 104.6, 48.5, 26.9, 24.2; LCMS *m/z* 187.1 [M+H]^+^.

##### 4.4.3.34 1-(5-Fluoroindolin-1-yl)ethan-1-one (**4s**)

Prepared by General Method 1.1 from 5-fluoroindoline. Isolated as an off-white solid (68 mg, 84%).

^1^H NMR (700 MHz, DMSO‑*d*_6_) *δ* 8.01 (dd, *J* = 8.8, 5.1 Hz, 1H), 7.09 (dd, *J* = 8.5, 2.3 Hz, 1H), 6.94 (td, *J* = 9.1, 2.6 Hz, 1H), 4.10 (t, *J* = 8.6 Hz, 2H), 3.14 (t, *J* = 8.5 Hz, 2H), 2.14 (s, 3H); ^13^C NMR (176 MHz, DMSO‑*d*_6_) *δ* 168.3, 158.2 (d, *J* = 238.8 Hz), 139.4, 134.3 (d, *J* = 8.7 Hz), 116.4 (d, *J* = 8.1 Hz), 112.9 (d, *J* = 22.6 Hz), 112.0 (d, *J* = 23.9 Hz), 48.4, 27.3, 23.8; LCMS *m/z* 180.0 [M+H]^+^.

##### 4.4.3.35 1-(5-Methoxyindolin-1-yl)ethan-1-one (**4t**)

Prepared by General Method 1.1 from 5-methoxy-2,3-dihydro-1*H*-indole hydrochloride. Isolated as an off-white solid (79 mg, 92%).

^1^H NMR (600 MHz, DMSO‑*d*_6_) *δ* 7.94 (d, *J* = 8.7 Hz, 1H), 6.83 (d, *J* = 2.7 Hz, 1H), 6.69 (dd, *J* = 8.7, 2.7 Hz, 1H), 4.07–4.01 (m, 2H), 3.70 (s, 3H), 3.10 (t, *J* = 8.5 Hz, 2H), 2.11 (s, 3H); ^13^C NMR (151 MHz, DMSO‑*d*_6_) *δ* 167.6, 155.5, 136.6, 133.4, 116.3, 111.6, 110.8, 55.3, 48.3, 27.6, 23.8; LCMS *m/z* 192.1 [M+H]^+^.

##### 4.4.3.36 1-(5-(Trifluoromethyl)indolin-1-yl)ethan-1-one (**4u**)

Prepared by General Method 1.1 from 5-(trifluoromethyl)-2,3-dihydro-1*H*-indole hydrochloride. Isolated as an off-white solid (90 mg, 87%).

^1^H NMR (600 MHz, DMSO‑*d*_6_) *δ* 8.16 (d, *J* = 8.4 Hz, 1H), 7.57 (s, 1H), 7.52 (d, *J* = 8.4 Hz, 1H), 4.15 (t, *J* = 8.7 Hz, 2H), 3.20 (t, *J* = 8.6 Hz, 2H), 2.19 (s, 3H); ^13^C NMR (151 MHz, DMSO‑*d*_6_) *δ* 169.5, 146.2, 133.2, 124.8 (q, *J* = 3.8 Hz), 124.6 (q, *J* = 271.4 Hz), 123.2 (q, *J* = 31.6 Hz), 121.8 (q, *J* = 3.6 Hz), 115.5, 48.6, 27.1, 24.1; LCMS *m/z* 230.1 [M+H]^+^.

##### 4.4.3.37 1-(5-Chloro-4-(trifluoromethyl)indolin-1-yl)ethan-1-one (**4v**)

*N*-Chlorosuccinimide (25 mg, 0.19mmol) was added to 1-[4-(trifluoromethyl)indolin-1-yl]ethanone (**4o**)(40 mg, 0.17 mmol) in MeCN (1 mL) and the reaction was heated to 65 °C for 18 h. Sat. aq. NaHCO_3_ (20 mL) was then added and stirred with CH_2_Cl_2_ (30 mL) for 5 min. The mixture was then passed through a TELOS phase separator and DMSO (1 mL) was added to the CH_2_Cl_2_ filtrate. The solvent was removed under reduced pressure the residue was purified by reverse phase chromatography (30g C18 Si eluting with 10–85% MeCN in H_2_O with 0.1% formic acid). Isolated as an off-white solid (27 mg, 59%).

^1^H NMR (700 MHz, CDCl_3_) *δ* 8.35 (d, *J* = 8.7 Hz, 1H), 7.32 (d, *J* = 8.7 Hz, 1H), 4.10 (t, *J* = 8.6 Hz, 2H), 3.41 (td, *J* = 8.7, 2.4 Hz, 2H), 2.24 (s, 3H); ^13^C NMR (176 MHz, CDCl_3_) *δ* 169.1, 142.9, 132.1, 131.2, 126.4, 124.6 (q, *J* = 31.5 Hz), 123.6 (q, *J* = 274.8 Hz), 120.3, 48.8, 28.5 (d, *J* = 4.0 Hz), 24.3; LCMS *m/z* 264.1 [M+H]^+^.

##### 4.4.3.38 1-(4,5-Dichloroindolin-1-yl)ethan-1-one (**4w**)

Prepared by General Method 1.1 from 4,5-dichloro-2,3-dihydro-1H-indole. Isolated as an off-white solid (95 mg, 92%).

^1^H NMR (700 MHz, DMSO‑*d*_6_) *δ* 7.96 (d, *J* = 8.6 Hz, 1H), 7.41 (d, *J* = 8.6 Hz, 1H), 4.19–4.13 (m, 2H), 3.18 (t, *J* = 8.5 Hz, 2H), 2.15 (s, 3H); ^13^C NMR (176 MHz, DMSO‑*d*_6_) *δ* 169.1, 143.1, 132.7, 129.1, 127.4, 124.2, 115.0, 48.3, 27.7, 23.8; LCMS *m/z* 230.0 [M+H]^+^.

##### 4.4.3.39 1-(6,7-Dichloroindolin-1-yl)ethan-1-one (**4x**)

Prepared by General Method 1.1 from 6,7-dichloro-2,3-dihydro-1H-indole. Isolated as an off-white solid (95 mg, 92%).

^1^H NMR (700 MHz, DMSO‑*d*_6_) *δ* 7.35 (d, *J* = 7.9 Hz, 1H), 7.26 (d, *J* = 7.9 Hz, 1H), 4.14 (t, *J* = 7.6 Hz, 2H), 3.05 (t, *J* = 7.6 Hz, 2H), 2.21 (s, 3H); ^13^C NMR (176 MHz, DMSO‑*d*_6_) *δ* 168.5, 142.4, 137.0, 130.1, 126.2, 123.8, 122.0, 51.5, 29.6, 23.4; LCMS *m/z* 230.1 [M+H]^+^.

##### 4.4.3.40 1-(4′-Chlorospiro[cyclopropane-1,3′-indolin]-1′-yl)ethan-1-one (**4y**)

Prepared by General Method 1.1 from 4′-chloro-1′,2′-dihydrospiro[cyclopropane-1,3′-indole] hydrochloride. Isolated as an off-white solid (76 mg, 82%).

^1^H NMR (700 MHz, CDCl_3_) *δ* 8.20 (d, *J* = 8.1 Hz, 1H), 7.06 (t, *J* = 8.1 Hz, 1H), 6.89 (d, *J* = 8.1 Hz, 1H), 3.97 (s, 2H), 2.18 (s, 3H), 1.95 (t, *J* = 5.7 Hz, 2H), 0.87 (t, *J* = 5.8 Hz, 2H); ^13^C NMR (176 MHz, CDCl_3_) *δ* 168.8, 145.6, 130.6, 128.2, 127.3, 125.3, 115.4, 58.7, 24.3, 23.8, 14.3; LCMS *m/z* 222.1 [M+H]^+^.

##### 4.4.3.41 1-(5′-Chlorospiro[cyclopropane-1,3′-indolin]-1′-yl)ethan-1-one (**4z**)

Prepared by General Method 1.1 from 5′-chloro-1′,2′-dihydrospiro[cyclopropane-1,3′-indole] hydrochloride. Isolated as an off-white solid (73 mg, 79%).

^1^H NMR (700 MHz, CDCl_3_) *δ* 8.13 (d, *J* = 8.6 Hz, 1H), 7.10 (dd, *J* = 8.6, 2.1 Hz, 1H), 6.58 (d, *J* = 1.8 Hz, 1H), 4.04 (s, 2H), 2.18 (s, 3H), 1.09 (t, *J* = 9.8 Hz, 4H).^13^C NMR (176 MHz, CDCl_3_) *δ* 168.7, 142.1, 138.7, 128.9, 127.0, 118.5, 117.6, 57.7, 24.1, 22.6, 18.0; LCMS *m/z* 222.1 [M+H]^+^.

##### 4.4.3.42 1-(4'-(Trifluoromethyl)spiro[cyclopropane-1,3′-indolin]-1′-yl)ethan-1-one (**4aa**)

Prepared by General Method 1.1 from 4'-(trifluoromethyl)spiro[cyclopropane-1,3′-indoline] hydrochloride. Isolated as an off-white solid (81 mg, 66%).

^1^H NMR (700 MHz, CDCl_3_) *δ* 8.53 (d, *J* = 7.7 Hz, 1H), 7.29 (d, *J* = 7.7 Hz, 1H), 7.26 (t, *J* = 8.0 Hz, 1H), 3.92 (s, 2H), 2.21 (s, 2H), 1.59 (s, 3H), 0.98–0.94 (m, 2H);^13^C NMR (176 MHz, CDCl_3_) *δ* 168.7, 146.2, 133.1, 127.4, 123.9 (q, *J* = 273.4 Hz), 123.6 (q, *J* = 31.3 Hz), 121.3 (q, *J* = 5.7 Hz), 120.1, 60.3, 24.4, 23.6, 16.9; LCMS *m/z* 256.1 [M+H]^+^.

##### 4.4.3.43 1-(4′-Chlorospiro[cyclopropane-1,3′-indolin]-1′-yl)-2-methylpropan-1-one (**5a**)

Prepared by General Method 1.1 from 4′-chloro-1′,2′-dihydrospiro[cyclopropane-1,3′-indole] hydrochloride and isobutyryl chloride. Isolated as an off-white solid (52 mg, 95%).

^1^H NMR (700 MHz, CDCl_3_) *δ* 8.27 (d, *J* = 7.2 Hz, 1H), 7.06 (t, *J* = 8.1 Hz, 1H), 6.89 (d, *J* = 8.1 Hz, 1H), 4.04 (s, 2H), 1.95 (q, *J* = 5.0 Hz, 2H), 1.21 (d, *J* = 6.7 Hz, 6H), 0.87 (q, *J* = 4.9 Hz, 2H); ^13^C NMR (176 MHz, CDCl3) *δ* 175.7, 146.1, 130.7, 128.2, 127.3, 125.3, 115.7, 57.9, 33.6, 23.8, 19.2, 14.2; LCMS *m/z* 250.1 [M+H]^+^.

##### 4.4.3.44 4′-Chloro-1'-(methylsulfonyl)spiro[cyclopropane-1,3′-indoline] (**5b**)

Pyridine (0.07 mL, 0.86 mmol) was added to a stirred suspension of 4′-chloro-1′,2′-dihydrospiro[cyclopropane-1,3′-indole] hydrochloride (62 mg, 0.29 mmol) in CH_2_Cl_2_ (2 mL) at 0 °C (external) and stirred until homogeneous. MeSO_2_Cl (0.03 mL, 0.43 mmol) was added dropwise, the ice bath was removed, and the mixture was stirred at RT for 1 h. The reaction was quenched by addition of water (10 mL) and then aq. HCl (5 mL, 1 M). The mixture was diluted with CH_2_Cl_2_ (20 mL) with vigorous stirring and then passed through a TELOS phase separator. The CH_2_Cl_2_ filtrate was concentrated, and the crude product purified by chromatography (10g Si eluting with 5–60% EtOAc in cyclohexane). Isolated as an off-white solid (61 mg, 0.23 mmol, 82%).

^1^H NMR (700 MHz, CDCl_3_) *δ* 7.35 (d, *J* = 8.1 Hz, 1H), 7.07 (t, *J* = 8.1 Hz, 1H), 6.93 (d, *J* = 8.1 Hz, 1H), 3.91 (d, *J* = 10.8 Hz, 2H), 2.90 (s, 3H), 1.90 (q, *J* = 4.9 Hz, 2H), 0.91 (q, *J* = 5.0 Hz, 2H); ^13^C NMR (176 MHz, CDCl_3_) *δ* 144.6, 131.1, 128.5, 128.4, 125.7, 112.6, 59.2, 35.0, 23.8, 13.8; LCMS *m/z* 258.9 [M+H]^+^.

##### 4.4.3.45 1-(4-Chloro-1*H*-indol-1-yl)ethan-1-one (**5c**)

Prepared by General Method 1.2 from 4-chloroindole. Isolated as a clear colourless oil (105 mg, 60%).

^1^H NMR (700 MHz, methanol-*d*_*4*_) *δ* 6.98–6.89 (m, 1H), 6.39 (t, *J* = 3.9 Hz, 1H), 5.94–5.77 (m, 2H), 5.38 (dd, *J* = 3.8, 0.7 Hz, 1H), 1.28 (s, 3H); ^13^C NMR (176 MHz, methanol-*d*_*4*_) *δ* 240.4, 206.8, 199.7, 197.5, 196.1, 195.9, 193.5, 185.4176.6, 93.1; LCMS *m/z* 192.1 [M − H]^-^.

##### 4.4.3.46 1-(4,5-Dichloro-1*H*-indol-1-yl)ethan-1-one (**5d**)

Prepared by General Method 1.2 from 4,5-dichloroindole. Isolated as an off-white solid (132 mg, 61%).

^1^H NMR (600 MHz, DMSO‑*d*_6_) *δ* 8.28 (d, *J* = 8.8 Hz, 1H), 8.07 (d, *J* = 3.8 Hz, 1H), 7.53 (dd, *J* = 7.1, 4.6 Hz, 1H), 6.81 (d, *J* = 3.8 Hz, 1H), 2.68 (s, 3H); ^13^C NMR (151 MHz, DMSO‑*d*_6_) *δ* 170.0, 133.8, 130.2, 130.0, 126.0, 125.7, 122.5, 115.9, 105.7, 23.8; LCMS *m/z* 227.0 [M − H]^-^.

##### 4.4.3.47 1-(8-Chloro-2,3-dihydro-4*H*-benzo[*b*][1,4]oxazin-4-yl)ethan-1-one (**5e**)

Prepared by General Method 1.1 from 8-chloro-3,4-dihydro-2*H*-1,4-benzoxazine. Isolated as an off-white solid (91 mg, 96%).

^1^H NMR (600 MHz, CDCl_3_) *δ* 7.19 (d, *J* = 7.5 Hz, 1H), 7.03 (s, 1H), 6.84 (t, *J* = 8.1 Hz, 1H), 4.43–4.35 (m, 2H), 3.96 (s, 2H), 2.31 (s, 3H); ^13^C NMR (151 MHz, CDCl_3_) *δ* 169.1, 143.3, 128.0, 127.1, 122.8, 122.3, 119.9, 67.9, 39.2, 22.9; LCMS *m/z* 212.1 [M+H]^+^.

##### 4.4.3.48 1,2,9,9a-Tetrahydro-3*H*-pyrrolo[1,2-*a*]indol-3-one (**5f**) [27]

Pd(OAc)_2_ (18 mg, 0.08 mmol), K_2_CO_3_ (326 mg, 2.36 mmol) and *N*-(2-allylphenyl)-2-bromo-acetamide (200 mg, 0.79 mmol) were added to a 10–20 mL thick-walled reaction vessel. The vessel was sealed with a Teflon-lined crimp cap, evacuated and backfilled with N_2_ (x3), and MeCN (8 mL) was added. The reaction mixture was stirred and heated at 40 °C overnight. The cooled mixture was diluted with CH_2_Cl_2_ (50 mL) and H_2_O (20 mL) and then passed through a TELOS phase separator. The CH_2_Cl_2_ filtrate was concentrated, and the crude product was purified by chromatography (25g Si eluting with 5–50% EtOAc in cyclohexane). Isolated as an off-white solid (105 mg, 77%).

^1^H NMR (700 MHz, CDCl_3_) *δ* 7.60 (d, *J* = 7.8 Hz, 1H), 7.21 (t, *J* = 7.7 Hz, 1H), 7.18 (d, *J* = 7.4 Hz, 1H), 7.03 (td, *J* = 7.5, 0.9 Hz, 1H), 4.64 (tdd, *J* = 10.0, 8.5, 6.1 Hz, 1H), 3.17 (dd, *J* = 15.5, 8.5 Hz, 1H), 2.92–2.80 (m, 2H), 2.59 (dd, *J* = 16.6, 8.4 Hz, 1H), 2.47 (ddd, *J* = 12.4, 7.7, 6.3 Hz, 1H), 1.99 (tdd, *J* = 12.4, 9.9, 8.6 Hz, 1H); ^13^C NMR (176 MHz, CDCl_3_) *δ* 171.8, 139.3, 134.2, 127.8, 125.3, 124.3, 114.9, 63.1, 36.5, 35.9, 29.5; LCMS *m/z* 174.1 [M+H]^+^.

##### 4.4.3.49 2,3-Dihydropyrrolo[1,2-*a*]indol-1-one (**5g)**[32]

*Step 1.* Indole (150 mg, 1.28 mmol), succinic anhydride (192 mg, 1.92 mmol) and CH_2_Cl_2_ (4 mL) were added to a 10–20 mL thick-walled reaction vessel, and the mixture stirred for 5 min to give a homogeneous solution. Et_3_N (0.54 mL, 3.84 mmol) and DMAP (15 mg, 0.13 mmol) were then added, the vessel was sealed with a Teflon-lined crimp cap, and stirred at RT overnight. DMSO (1.5 mL) was added and the CH_2_Cl_2_ removed under vacuo. The DMSO solution was directly purified by reverse phase chromatography (60g C18 Si eluting with 15–75% MeCN in H_2_O with 0.1% formic acid) to give 4-indol-1-yl-4-oxo-butanoic acid (259 mg, 93%) as a light brown solid.

^1^H NMR (700 MHz, DMSO‑*d*_6_) *δ* 12.25 (s, 1H), 8.32 (d, *J* = 8.2 Hz, 1H), 7.93 (d, *J* = 3.8 Hz, 1H), 7.61 (d, *J* = 7.7 Hz, 1H), 7.34–7.28 (m, 1H), 7.28–7.23 (m, 1H), 6.75 (d, *J* = 3.7 Hz, 1H), 3.28–3.24 (m, 2H), 2.68–2.63 (m, 2H); ^13^C NMR (176 MHz, DMSO‑*d*_6_) *δ* 173.6, 171.1, 134.9, 130.2, 126.3, 124.6, 123.4, 120.9, 115.8, 108.4, 30.2, 28.2; LCMS *m/z* 172.1 [M+H]^+^.

*Step 2.* 4-Indol-1-yl-4-oxo-butanoic acid (245 mg, 1.13 mmol) and *N*-hydroxypthalimide (202 mg, 1.24 mmol) were added to a 10–20mL thick-walled reaction vessel, and sealed with a Teflon-lined crimp cap. THF (6 mL) and then *N,N*-diisopropylcarbodiimide (0.19 mL, 1.24 mmol) were added, and the resulting solution was stirred for 18 h at RT. The mixture was diluted with CH_2_Cl_2_ (30 mL), H-MN dry loading support was added, and the solvent was removed in vacuo. Purification by chromatography (50g Si eluting with 0–5% EtOAc in CH_2_Cl_2_) gave (1,3-dioxoisoindolin-2-yl)-4-indol-1-yl-4-oxo-butanoate (349 mg, 0.96 mmol, 85%) as an off-white solid.

^1^H NMR (600 MHz, DMSO‑*d*_6_) *δ* 8.35 (d, *J* = 8.2 Hz, 1H), 8.01–7.92 (m, 5H), 7.63 (d, *J* = 7.7 Hz, 1H), 7.37–7.31 (m, 1H), 7.31–7.25 (m, 1H), 6.78 (d, *J* = 3.8 Hz, 1H), 3.55–3.48 (m, 2H), 3.25–3.18 (m, 2H); ^13^C NMR (151 MHz, DMSO‑*d*_6_) *δ* 170.0, 169.6, 161.7, 135.5, 134.9, 130.2, 128.2, 126.3, 124.7, 124.03, 123.6, 121.0, 115.9, 108.7, 29.8, 25.3.

*Step 3.* Tris(2-phenylpyridine)iridium (23 mg, 0.03 mmol) and (1,3-dioxoisoindolin-2-yl) 4-indol-1-yl-4-oxo-butanoate (250 mg, 0.69 mmol) were added to a 10–20 mL thick-walled reaction vessel, and sealed with a Teflon-lined crimp cap. Anhydrous DMSO (10 mL) was added and the vessel purged with N_2_ (x3). The reaction vessel was illuminated with blue LED light irradiation (455 nm) with stirring at RT for 18 h. The reaction mixture was diluted in Et_2_O (25 mL), and then washed with water (2 × 25 mL), aq. NaOH (2 M, x2) and brine. The organic layer was dried over Na_2_SO_4_ and concentrated in vacuo. The crude product was purified by column chromatography on silica gel (eluting with 70–0% petroleum ether in CH_2_Cl_2_). Isolated as an off-white solid (41 mg, 35%).

^1^H NMR (300 MHz, CDCl_3_) *δ* 8.14–8.01 (m, 1H), 7.64–7.44 (m, 1H), 7.30–7.24 (m, 2H), 6.28 (d, *J* = 0.7 Hz, 1H), 3.20–3.14 (m, 2H), 3.12–3.06 (m, 2H); ^13^C NMR (75 MHz, CDCl_3_) *δ* 171.8, 143.7, 135.4, 130.5, 124.1, 123.3, 120.6, 113.7, 100.5, 34.9, 19.7; LCMS *m/z* 172.1 [M+H]^+^.

##### 4.4.3.50 9,9a-Dihydro-1*H*,3*H*-oxazolo[3,4-*a*]indol-3-one (**5h**)

A solution of triphosgene (80 mg, 0.27 mmol) in CH_2_Cl_2_ (1.0 mL) was added dropwise to a suspension of (2,3-dihydro-1*H*-indol-2-yl)methanol (100 mg, 0.67 mmol) and DIPEA (230 μL, 1.34 mmol) in CH_2_Cl_2_ (10 mL) at 0 °C (external), and the reaction mixture was then stirred at RT for 2 h. Sat. aq. NaHCO_3_ (10 mL) was cautiously added. The organic phase was separated, dried, and concentrated under reduced pressure. The crude product was purified by chromatography (25g Si eluting with 10–60% EtOAc in cyclohexane). Isolated as an off-white solid (104 mg, 89%).

^1^H NMR (600 MHz, DMSO‑*d*_6_) *δ* 7.30–7.22 (m, 3H), 7.09 (td, *J* = 7.3, 1.5 Hz, 1H), 4.86 (app p, *J* = 8.7 Hz, 1H), 4.76 (t, *J* = 8.5 Hz, 1H), 4.37 (t, *J* = 8.3 Hz, 1H), 3.18 (ddd, *J* = 45.8, 16.0, 9.1 Hz, 2H); ^13^C NMR (151 MHz, DMSO‑*d*_6_) *δ* 156.5, 140.6, 133.7, 127.5, 125.5, 124.5, 114.6, 70.9, 58.9, 34.9; LCMS *m/z* 176.0 [M+H]^+^.

##### 4.4.3.51 10,10a-Dihydro-1*H*-[1,4]oxazino[4,3-*a*]indol-4(3*H*)-one (**5i**)

(2,3-Dihydro-1H-indol-2-yl)methanol (110 mg, 0.73 mmol) was added to a stirred, suspension of *t*-BuOK (83 mg, 0.74 mmol) in THF (1.0 mL) at RT under argon. The mixture was then warmed to 40 °C, ethyl chloroacetate (80 μL, 0.73 mmol) was added, and stirred for an additional 1 h. The cooled mixture was concentrated under reduced pressure, H_2_O (2.0 mL) was added, and extracted with CH_2_Cl_2_ (10 mL). The organic phase was dried, concentrated under reduced pressure and the crude product was purified by reverse phase chromatography (30g C18 Si eluting with 10–85% MeCN in H_2_O with 0.1% formic acid). Isolated as a yellow gum (30 mg, 22%).

^1^H NMR (600 MHz, DMSO‑*d*_6_) *δ* 7.84 (d, *J* = 7.9 Hz, 1H), 7.32 (d, *J* = 7.4 Hz, 1H), 7.22 (t, *J* = 7.7 Hz, 1H), 7.08 (t, *J* = 7.4 Hz, 1H), 4.44–4.36 (m, 1H), 4.29–4.24 (m, 2H), 4.08 (d, *J* = 16.6 Hz, 1H), 3.70 (t, *J* = 10.9 Hz, 1H), 3.10 (dd, *J* = 15.4, 8.1 Hz, 1H), 2.85 (dd, *J* = 15.3, 11.5 Hz, 1H); ^13^C NMR (151 MHz, DMSO‑*d*_6_) *δ* 164.7, 141.6, 130.7, 127.2, 125.1, 124.5, 116.3, 67.4, 66.3, 58.6, 30.9; LCMS *m/z* 190.1 [M+H]^+^.

##### 4.4.3.52 7-Chloro-1,2,9,9a-tetrahydro-3*H*-pyrrolo[1,2-*a*]indol-3-one (**5j**)

*N*-Chlorosuccinimide (44 mg, 0.33mmol) was added to 2,3,3a,4-tetrahydropyrrolo[1,2-*a*]indol-1-one (**5f**) (54 mg, 0.31 mmol) in MeCN (2 mL) and the reaction was heated to 65 °C for 18 h. Sat. aq. NaHCO_3_ (20 mL) was added and stirred with CH_2_Cl_2_ (30 mL) for 5 min. The mixture was passed through a TELOS phase separator, DMSO (1 mL) was added and the CH_2_Cl_2_ removed under reduced pressure. The residue was purified by reverse phase chromatography (30g C18 Si eluting with 10–85% MeCN in H_2_O with 0.1% formic acid). Isolated as an off-white (23 mg, 36%).

^1^H NMR (300 MHz, CDCl_3_) *δ* 7.51 (d, *J* = 8.2 Hz, 1H), 7.23–7.10 (m, 2H), 4.66 (tdd, *J* = 10.0, 8.5, 6.1 Hz, 1H), 3.17 (dd, *J* = 15.9, 8.5 Hz, 1H), 2.96–2.75 (m, 2H), 2.54 (dddd, *J* = 14.0, 13.3, 7.8, 3.4 Hz, 2H), 2.00 (tdd, *J* = 12.4, 9.9, 8.6 Hz, 1H); ^13^C NMR (75 MHz, CDCl_3_) *δ* 171.9, 137.9, 136.1, 129.2, 127.8, 125.7, 115.6, 63.2, 36.3, 35.8, 29.5; LCMS *m/z* 208.0 [M+H]^+^.

##### 4.4.3.53 8-Chloro-1,2,9,9a-tetrahydro-3*H*-pyrrolo[1,2-*a*]indol-3-one (**5k**)

In a 10–20 mL thick-walled reaction vessel, K_2_CO_3_ (293 mg, 2.12 mmol), *N*-(2-allyl-3-chloro-phenyl)-2-bromoacetamide (204 mg, 0.71 mmol) and Pd(OAc)_2_ (16 mg, 0.07 mmol) were added, the vessel was sealed with a Teflon-lined crimp cap, and backfilled with N_2_ (x3). MeCN (7.5 mL) was added, and the reaction mixture was stirred at 40 °C overnight. The mixture was cooled to RT, diluted with CH_2_Cl_2_ (50 mL) and water (20 mL), passed through a TELOS phase separator and the solvent evaporated in vacuo. The residue was purified by chromatography (25g Si eluting with 5–60% EtOAc in cyclohexane). Isolated as an off-white solid (42 mg, 29%)

^1^H NMR (700 MHz, CDCl_3_) *δ* 7.49 (d, *J* = 7.8 Hz, 1H), 7.16 (t, *J* = 7.9 Hz, 1H), 7.01 (d, *J* = 8.1 Hz, 1H), 4.70–4.63 (m, 1H), 3.27 (dd, *J* = 16.2, 8.7 Hz, 1H), 2.91–2.79 (m, 2H), 2.59 (dd, *J* = 16.6, 8.4 Hz, 1H), 2.54–2.48 (m, 1H), 2.02 (tt, *J* = 12.4, 9.4 Hz, 1H); ^13^C NMR (176 MHz, CDCl_3_) *δ* 172.2, 140.6, 132.5, 130.9, 129.3, 124.2, 113.0, 62.5, 36.2, 35.2, 29.6; LCMS *m/z* 208.1 [M+H]^+^.

##### 4.4.3.54 3a-Methyl-3,4-dihydro-2*H*-pyrrolo[1,2-*a*]indol-1-one (**5l**) [33]

To an oven-dried 10–20mL thick-walled reaction vessel were added Pd(OAc)_2_ (15 mg, 0.07 mmol) and anhydrous Na_2_CO_3_ (105 mg, 0.99 mmol), the vessel was sealed with a Teflon-lined crimp cap, and backfilled with N_2_ (x3). Anhydrous 1,4-dioxane (3 mL) was added and the mixture stirred at RT until homogeneous. Ethyl nicotinate (0.04 mL, 0.26 mmol) was then added and the mixture stirred for 10 min. A solution of 4-methyl-*N*-phenyl-pent-4-enamide (125 mg, 0.66 mmol) in 1,4-dioxane (3 mL) was added, the vessel was evacuated and refilled with O_2_, and heated to 70 °C for 24 h. The reaction mixture was cooled, filtered through a short pad of silica gel with EtOAc as eluent and the filtrate concentrated in vacuo. The residue was purified by chromatography (25 g SNAP KP Si cartridge eluting with 2–45% EtOAc in cyclohexane). Isolated as an off-white solid (54 mg, 44%).

^1^H NMR (600 MHz, CDCl_3_) *δ* 7.60 (d, *J* = 7.8 Hz, 1H), 7.26–7.16 (m, 2H), 7.04 (td, *J* = 7.5, 0.7 Hz, 1H), 3.08 (d, *J* = 15.5 Hz, 1H), 2.98–2.85 (m, 2H), 2.56 (ddd, *J* = 16.9, 7.7, 1.7 Hz, 1H), 2.26–2.13 (m, 2H), 1.38 (s, 3H); ^13^C NMR (151 MHz, CDCl_3_) *δ* 171.7, 138.4, 133.5, 127.8, 125.7, 124.3, 115.5, 69.5, 43.49, 35.3, 35.1, 25.6; LCMS *m/z* 188.1 [M+H]^+^.

##### 4.4.3.55 2,2-Difluoro-1,2,9,9a-tetrahydro-3H-pyrrolo[1,2-*a*]indol-3-one (**5m**) [28]

To a 10–20 mL thick-walled reaction vessel, 2-allylaniline (137 mg, 1.03 mmol), 1,4-dioxane (7.5 mL) and 1,4-dimethylpiperazine (0.42 mL, 3.09 mmol) were added, the vessel was sealed with a Teflon-lined crimp cap, and backfilled with N_2_ (x3). Ethyl iododifluoroacetate (0.45 mL, 3.09 mmol) was then added and the solution stirred at 80 °C for 20 h. The solvents were removed in vacuo and the residue purified directly by chromatography (50 g Si eluting with 5–60% EtOAc in cyclohexane). Isolated as an off-white solid (115 mg, 53%).

^1^H NMR (700 MHz, CDCl_3_) *δ* 7.66 (d, *J* = 7.8 Hz, 1H), 7.28 (t, *J* = 7.7 Hz, 1H), 7.26–7.24 (m, 1H), 7.14 (t, *J* = 7.5 Hz, 1H), 4.68–4.60 (m, 1H), 3.31 (dd, *J* = 15.5, 8.2 Hz, 1H), 3.05–2.94 (m, 2H), 2.47–2.35 (m, 1H); ^13^C NMR (176 MHz, CDCl_3_) *δ* 159.44 (dd, *J* = 33.5, 28.2 Hz), 138.04, 133.11, 128.39, 126.14, 125.58, 120.91 (dd, *J* = 258.8, 253.1 Hz), 115.83, 56.81 (d, *J* = 6.4 Hz), 39.36 (dd, *J* = 24.8, 21.1 Hz), 35.52;^19^F NMR (659 MHz, CDCl_3_) *δ* −105.18 (dd, *J* = 267.7, 12.4 Hz), −105.98 (ddd, *J* = 267.7, 27.0, 13.8 Hz); LCMS *m/z* 210 [M+H]^+^.

##### 4.4.3.56 4,5-Dichloroindoline (**5n**)

Purchased from Enamine, EN300-1178090.

##### 4.4.3.57 4-Chloro-1-ethylindoline hydrochloride (**5o**)

DIPEA (1.1 mL, 6.3 mmol) and EtI (240 μL, 3.0 mmol) were added to a stirred solution of 4-chloro-2,3-dihydro-1*H*-indole hydrochloride (384 mg, 2.5 mmol) in DMF (0.3 mL) at RT and the reaction was then heated to 80 °C for 16 h. The reaction mixture was diluted with EtOAc (10 mL) and sat. brine (10 mL), the organic phase was dried (Na_2_SO_4_), and concentrated under reduced pressure. The crude material was purified by reverse phase chromatography to give **5o** as an oil. The free base was treated with HCl (3.0 mL, 3 M in cyclopentyl methyl ether) at RT for 5 min, and then concentrated under reduced pressure to give the hydrochloride salt. Isolated as an off-white solid (107 mg, 20%).

^1^H NMR (700 MHz, CDCl_3_) *δ* 7.43–7.35 (m, 3H), 3.91 (s, 2H), 3.50 (d, *J* = 6.0 Hz, 2H), 3.33 (t, *J* = 7.4 Hz, 2H), 1.45 (t, *J* = 7.1 Hz, 3H); ^13^C NMR (176 MHz, CDCl_3_) *δ* 141.0, 134.1, 132.2, 130.4, 130.4, 117.8, 52.1, 51.7, 28.1, 9.3; LCMS *m/z* 182.1 [M+H]^+^.

## Funding

The 10.13039/501100000765ARUK UCL Drug Discovery Institute is core funded by 10.13039/501100002283Alzheimer's Research UK (grant number 560832). Y.Z. and E.Y.J. are supported by 10.13039/501100000289Cancer Research UK (C375/A17721), the 10.13039/501100000265UK Medical Research Council (MR/T000503/1), and the 10.13039/100010269Wellcome Trust (223133/Z/21/Z).

## Declaration of competing interest

B.N.A., N.J.W, Y.Z., E.Y.J. and P.V.F. are co-inventors of patent application #WO 2020043866 issued to UCL Business Ltd, which describes inhibitors of Notum.

## Data Availability

Data will be made available on request.
