# Supplementary material for: Designed switch from covalent to non-covalent inhibitors of carboxylesterase Notum activity
Source: Eur J Med Chem. 2023 May 5;251:115132. doi: 10.1016/j.ejmech.2023.115132 (PMC10626578; doi:10.1016/j.ejmech.2023.115132)
Supplement: Multimedia component 1 [file mmc1.docx]

**SUPPLEMENTARY MATERIALS**

**Designed Switch from Covalent to Non-Covalent Inhibitors of Carboxylesterase Notum Activity**

Benjamin N. Atkinson,^a^ Nicky J. Willis,^a^ Yuguang Zhao,^b^ Chandni Patel,^a^ Sarah Frew,^a^ Kathryn Costelloe,^a^ Lorenza Magno,^a^ Fredrik Svensson,^a,^* E. Yvonne Jones,^b^ and Paul V. Fish^a^

^a^ Alzheimer’s Research UK UCL Drug Discovery Institute, University College London, The Cruciform Building, Gower Street, London, WC1E 6BT, U.K.

^b^ Division of Structural Biology, Wellcome Centre for Human Genetics, University of Oxford, The Henry Wellcome Building for Genomic Medicine, Roosevelt Drive, Oxford, OX3 7BN, U.K.

* Corresponding author. FS, Phone: +44 (0)20 7679 0811; E-mail: [f.svensson@ucl.ac.uk](mailto:f.svensson@ucl.ac.uk).

**Table of contents:**

Page S3 ADME protocols and results (**Tables S1-S5**)

Page S5 Metabolite identification studies of **4y** in MLM (**Tables S6-S7**)

Page S7 Electron density omit maps and overlay of **3a**, **3b** and **3c** (**Figure S1**)

Page S8 Overlay of **3a**, **3b** and **3c** (**Figure S2**)

Page S9 Spectroscopic and analytical data for 1-(4,5-dichloroindolin-1-yl)ethan-1-one (**4w**) (**Figures S3**)

Page S11 Notum OPTS and TCF/LEF screening data concentration-response curves for **4w** (**Figures S4**)

Page S14 SM references

**ADME protocols and results**

*In vitro* ADME studies reported in this work were performed by WuXi AppTec (Shanghai, China).

WuXi AppTec: <https://labtesting.wuxiapptec.com/dmpk-services/in-vitro-adme/>

1. **Distribution coefficient (LogD_7.4_):**

Representative assay protocols have been described in detail.^1,2^

**Table S1**: Results

| **Compound** | **LogD @ pH 7.40** |
| --- | --- |
| **4w** | 3.1 |

1. **Aqueous solubility at pH 7.4:**

Representative assay protocols have been described in detail. ^1,2^

**Table S2**: Results

| **Compound** | **Thermodynamic Solubility @ RT**  (μg/mL) (n = 2) |
| --- | --- |
| **4v** | 1.55 |
| **4w** | < 0.36 * |
| **4y** | 0.186 |
| **5d** | < 0.36 * |
| **5f** | 577 |

* below lower limit of quantification

1. **Permeability using MDR1-MDCKII cell monolayer:**

Representative assay protocols have been described in detail. ^1,2^

**Table S3:** Results

| **Compound** | **MDR1-MDCKII**  Mean (n=2) | | | | |
| --- | --- | --- | --- | --- | --- |
|  | **Apical to Basal**  *P*_app_ (10^-6^ cm/sec) | **Basal to Apical**  *P*_app_ (10^-6^ cm/sec) | **Efflux Ratio** | **A to B**  **% Recovery** | **B to A**  **% Recovery** |
| **4w** | 35.5 | 21.1 | 0.59 | 75 | 81 |

1. **Stability in liver microsomes:**

Representative assay protocols have been described in detail. ^1,2^

**Table S4**: Results

| **Compound** | **MLM Stability** | |
| --- | --- | --- |
|  | **t_1/2_**  (min) | **Cl_i_** (μL/min/mg protein) |
| **3a** | 8 | 180 |
| **4d** | 3.8 | 360 |
| **4v** | 2.1 | 650 |
| **4w** | 1.2 | 1150 |
| **4y** | 1.5 | 895 |
| **4z** | 2.2 | 620 |
| **4aa** | 2.0 | 670 |
| **5b** | 7.0 | 200 |
| **5d** | 1.1 | 1320 |
| **5f** | 19 | 73 |
| **5m** | 16 | 91 |
| **Compound** | **HLM Stability** | |
|  | **t_1/2_**  (min) | **Cl_i_** (μL/min/mg protein) |
| **4g** | 2.2 | 640 |
| **4o** | 1.5 | 900 |
| **4w** | 10 | 138 |

1. **Stability in plasma:**

Representative assay protocols have been described in detail. ^1,2^

**Table S5**: Results

| **Compound** | **Mouse plasma Stability** |
| --- | --- |
|  | **t_1/2_** (min) |
| **4w** | >280 |

**Metabolite identification studies of 4y in MLM**

The test compound **4y** at 10 µM was incubated with mouse liver microsomes (MLM) at 37 ^o^C for 60 min. The positive control, 7-ethoxycoumarin (7-EC) at 10 µM, was run concurrently to assess Phase I metabolic activities in liver microsomes. The results indicated that liver microsomes incubation system was reliable for metabolic study. After incubation, the samples were analysed by LC-UV-MS. The structures of the metabolites were proposed based on the interpretation of their MS and MS^2^ data.

In addition to unchanged **4y** (MW = 221.68), a total of four metabolites of **4y** were detected and identified by LC-UV-MS*^n^* (*n* = 1 & 2) from mouse liver microsomes (**Table S6**). The metabolites were assigned as below: M1: Di-oxygenation metabolite (MW = 253.68, P + 2O); M2-M3: Mono-oxygenation metabolites (MW = 237.68, P + O); M4: Amide hydrolysis and dehydrogenation metabolite (MW = 177.63, P – COCH_2_ – 2H)_._

The relative abundance of **4y** and each of the putative metabolites were calculated using peak area (peak area under the UV spectrum at 250-280 nm) of individual metabolite relative to the total area of all detected drug‑related components (**Table S6**).

The accurate mass measurements of these compounds are presented in **Table S7**.

In mouse liver microsomes, **4y** and 4 metabolites (M1-M4) were detected. M4 was considered to be the primary metabolite with relative abundance of 61%. The relative abundance of the other metabolites was less than 9%.

The parent, **4y**, accounted for 30% of the total drug‑related components in mouse liver microsomes after incubation at 37 °C for 60 min.

The proposed metabolic pathway of **4y** in mouse liver microsomes was amide hydrolysis, dehydrogenation and mono-oxygenation (**Figure S1**).

Table S6: Summary of 4y and its metabolites in mouse liver microsomes

| **Code** | [M + H]^+^*m/z* | **RT**  **(min)** | **Relative Abundance**  **(UV peak area %Total)** | **Metabolic Pathways** | |
| --- | --- | --- | --- | --- | --- |
|  |  |  |  | |  |
| **M1** | 254.0569 | 11.23 | + | Di-oxygenation (P + 2O) | |
| **M2** | 238.0624 | 14.10 | + | Mono-oxygenation (P + O) | |
| **M3** | 238.0624 | 16.45 | 8.20 | Mono-oxygenation (P + O) | |
| **M4** | 178.0414 | 18.12 | 61.32 | Amide hydrolysis and dehydrogenation (P – COCH_2_ – 2H) | |
| **4y** | 222.0676 | 21.10 | 30.48 | Parent | |

Note: RT: Retention time of LC-MS.

+: Only detected by MS, but too weak to be integrated under the UV wavelength at 250-280 nm;

Table S7: Accurate mass measurements of 4y and its metabolites in mouse liver microsomes.

| **Code** | **RT**  **(min)** | **Formula** | **Exact Mass** | **Theoretical Mass (*m/z*)** | **Measured Mass (*m/z*)** | **Mass Error (ppm)** | **Source** |
| --- | --- | --- | --- | --- | --- | --- | --- |
|  |  |  |  |  |  |  |  |
| **M1** | 11.23 | C12H12ClNO3 | 253.0506 | 254.0578 | 254.0569 | -3.5 | *MLM |
| **M2** | 14.10 | C12H12ClNO2 | 237.0557 | 238.0629 | 238.0624 | -2.1 | *MLM |
| **M3** | 16.45 | C12H12ClNO2 | 237.0557 | 238.0629 | 238.0624 | -2.1 | *MLM |
| **M4** | 18.12 | C10H9ClN | 177.0345 | 178.0418 | 178.0414 | -2.2 | *MLM |
| **4y** | 21.10 | C12H12ClNO | 221.0607 | 222.0680 | 222.0676 | -1.8 | *Std, MLM |

Exact Mass: Monoisotopic Mass; Theoretical Mass (*m/z*) = Exact Mass + 1.0073; Theoretical Mass (*m*/*z*) = Exact Mass - 1.0073; RT: Retention time of LC-MS; Std: Standard compound; *: The species which the data listed in this table were derived from.

**Figure S1: Electron density omit maps**

**Figure S1.** Electron density |F_O_ - F_C_| omit maps contoured to 3σ (green mesh).

| 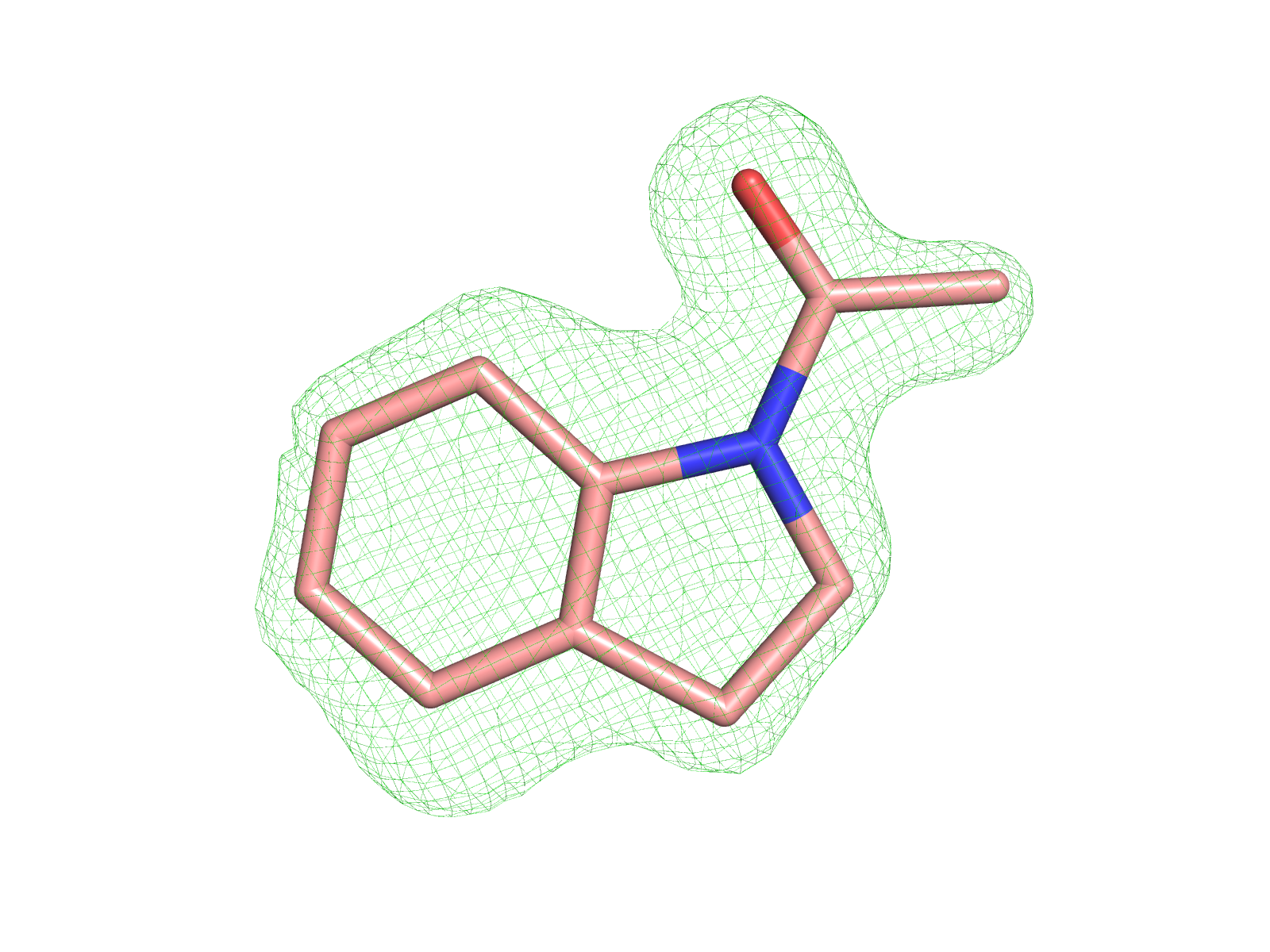 | | 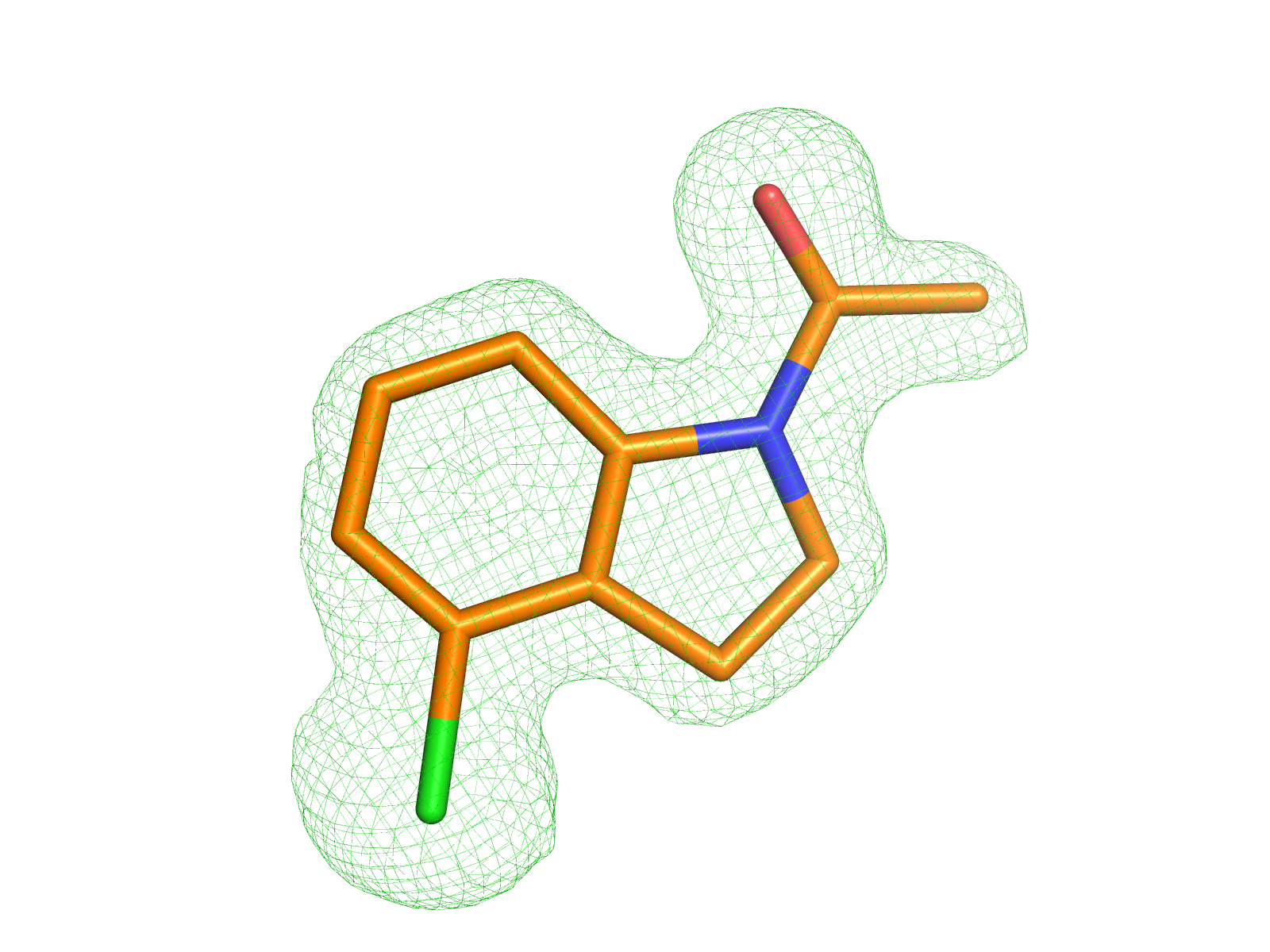 | |
| --- | --- | --- | --- |
|  | **3a**  PDB 8BT8 |  | **4g**  PDB 8BTA |
| 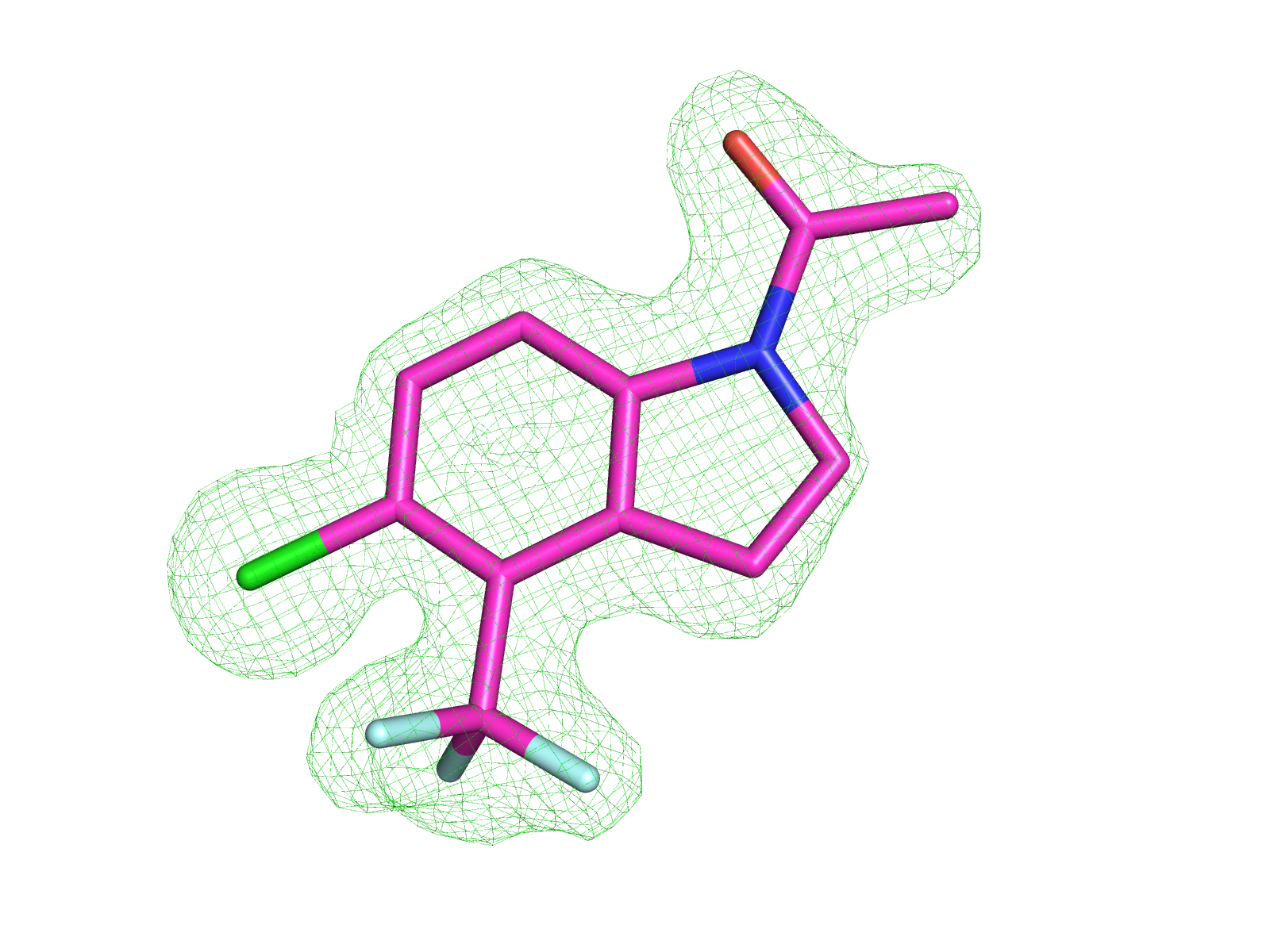 | | 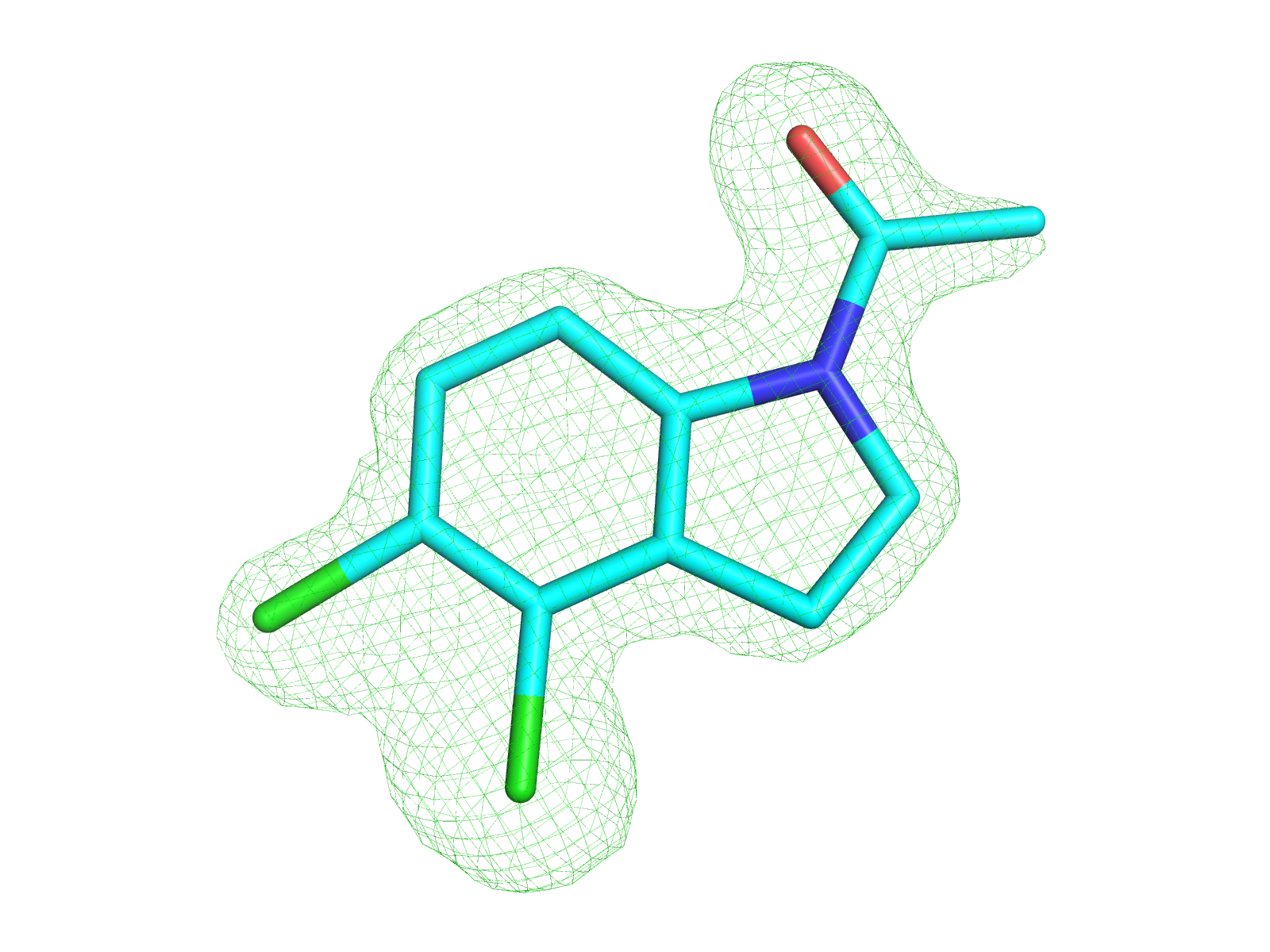 | |
|  | **4v**  PDB 8BT2 |  | **4w**  PDB 8BTC |
| 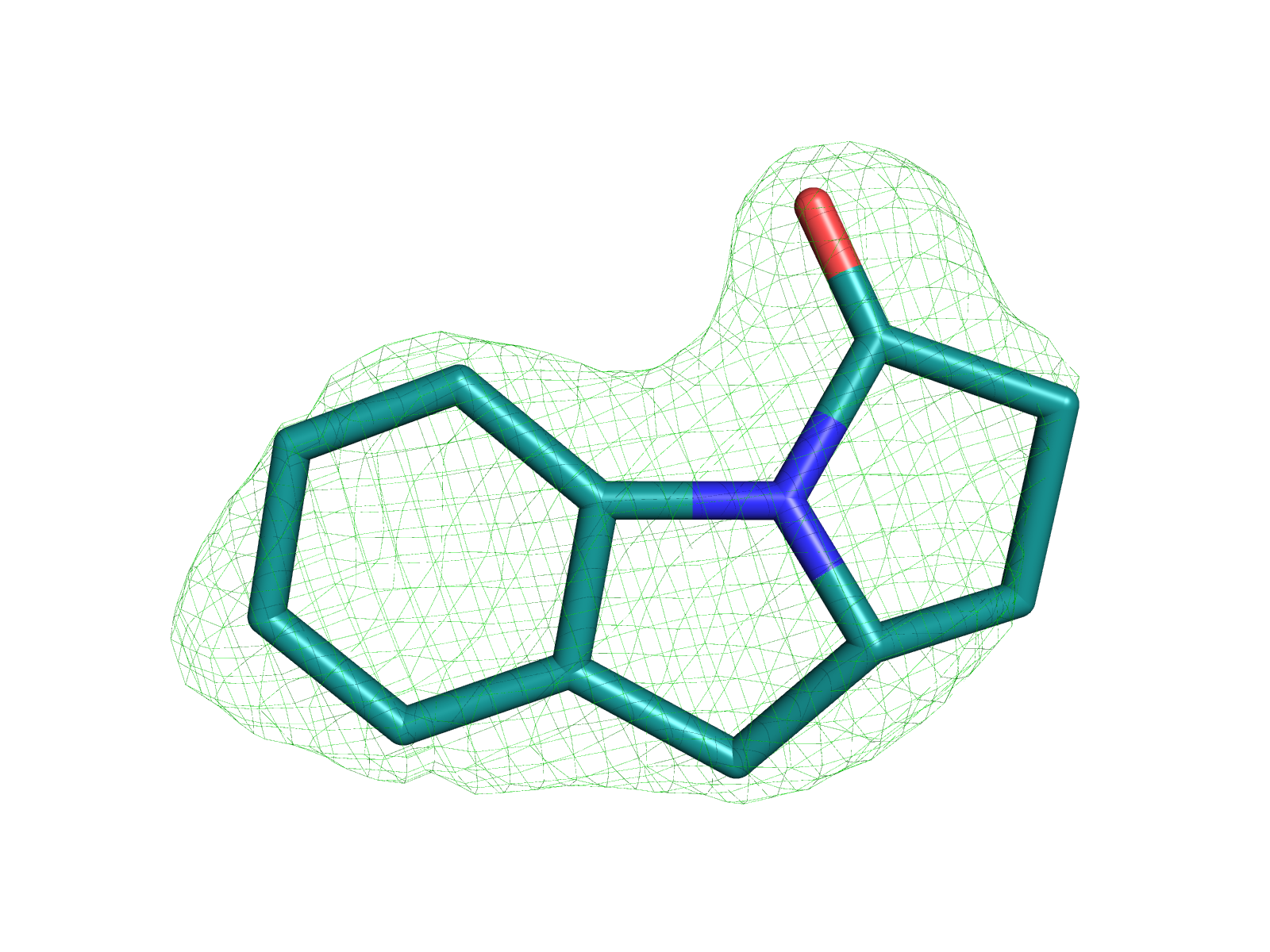 | | 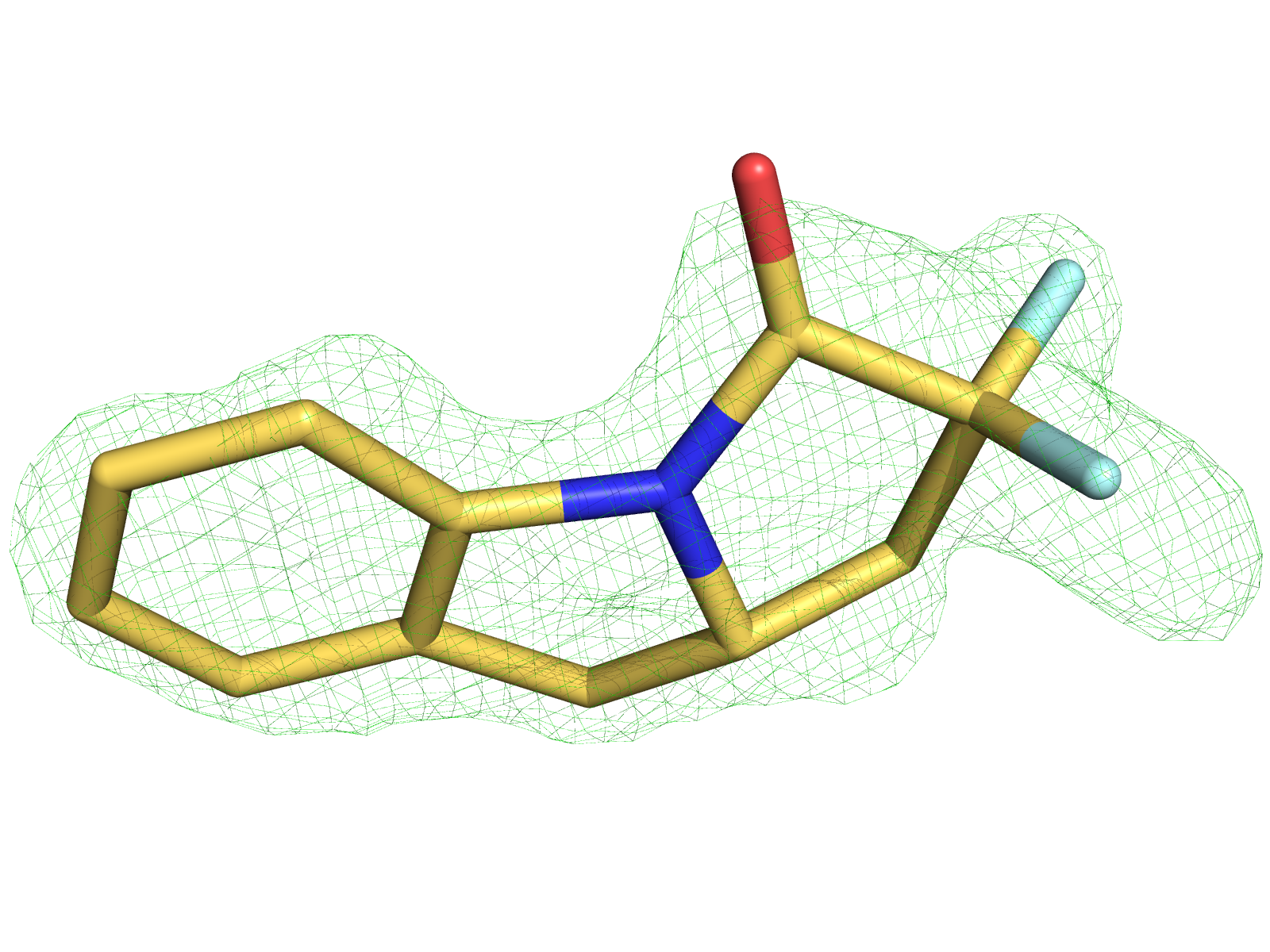 | |
|  | **5f**  PDB 8BSZ |  | **5m**  PDB 8BT0 |

**Figure S2.** **Overlay of 3a, 3b and 3c.**


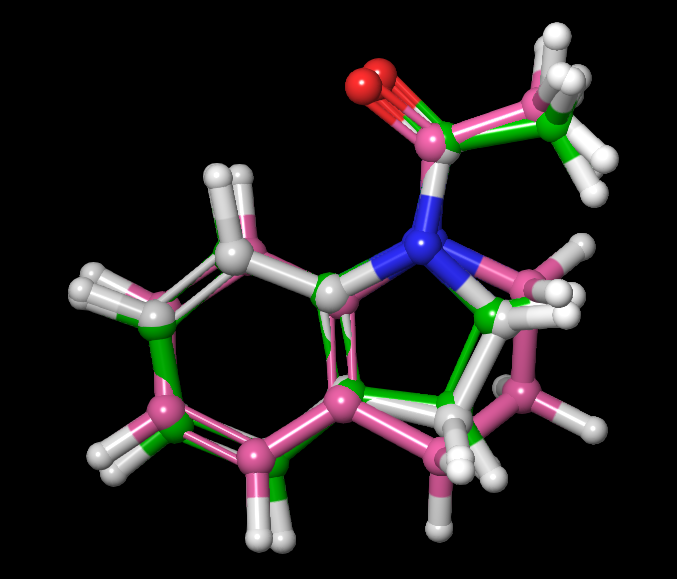


Overlay of indoline **3a** (white), indole **3b** (green) and tetrahydroquinoline **3c** (pink) assuming they all bind to Notum in a similar orientation to **3a** (PDB 8BTA).

**Figure S3: Spectroscopic and analytical data for 1-(4,5-dichloroindolin-1-yl)ethan-1-one (4w)**


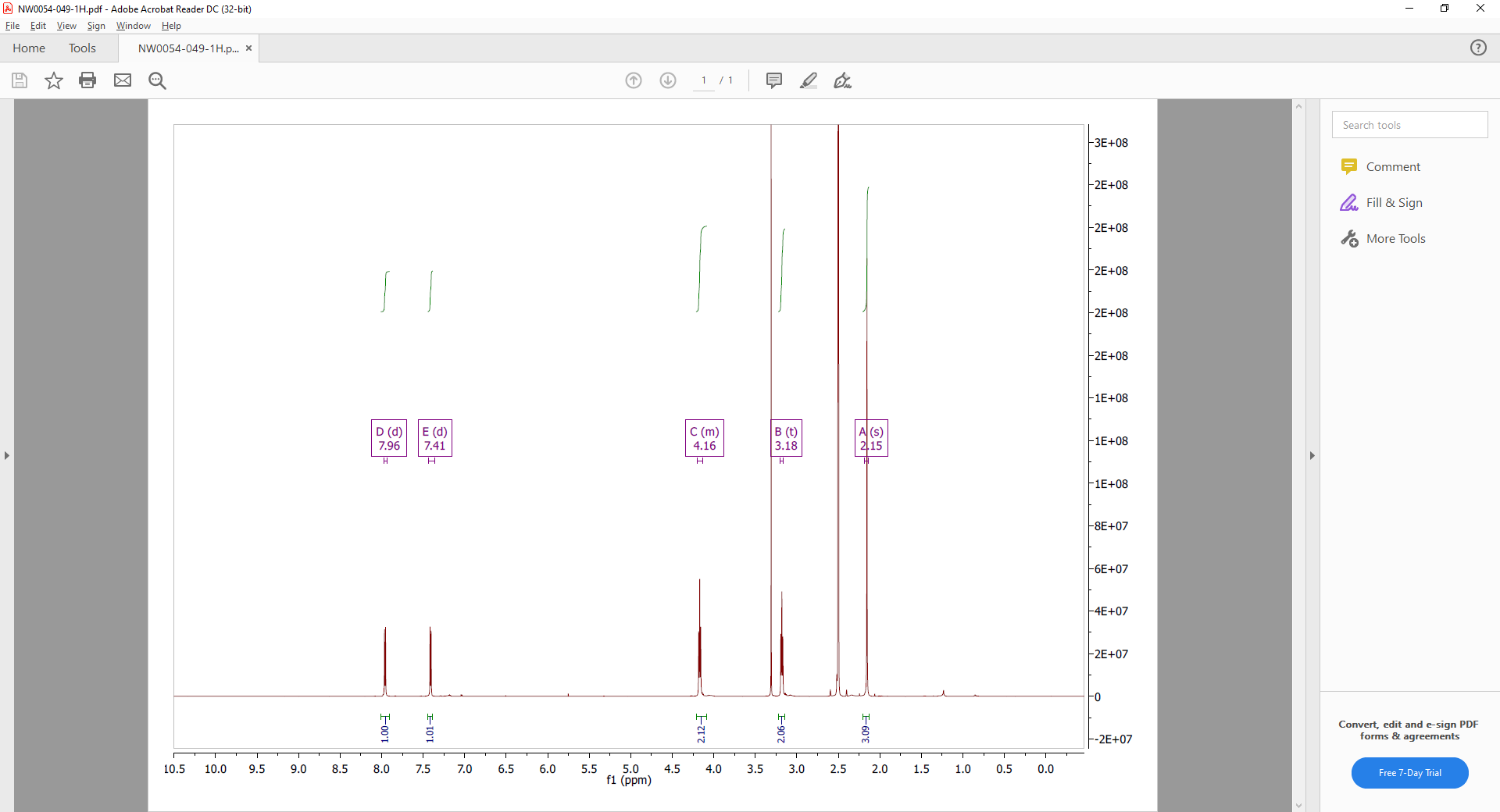


**Figure S3A**. ^1^H NMR (700 MHz, DMSO-*d_6_*) for **4w**.


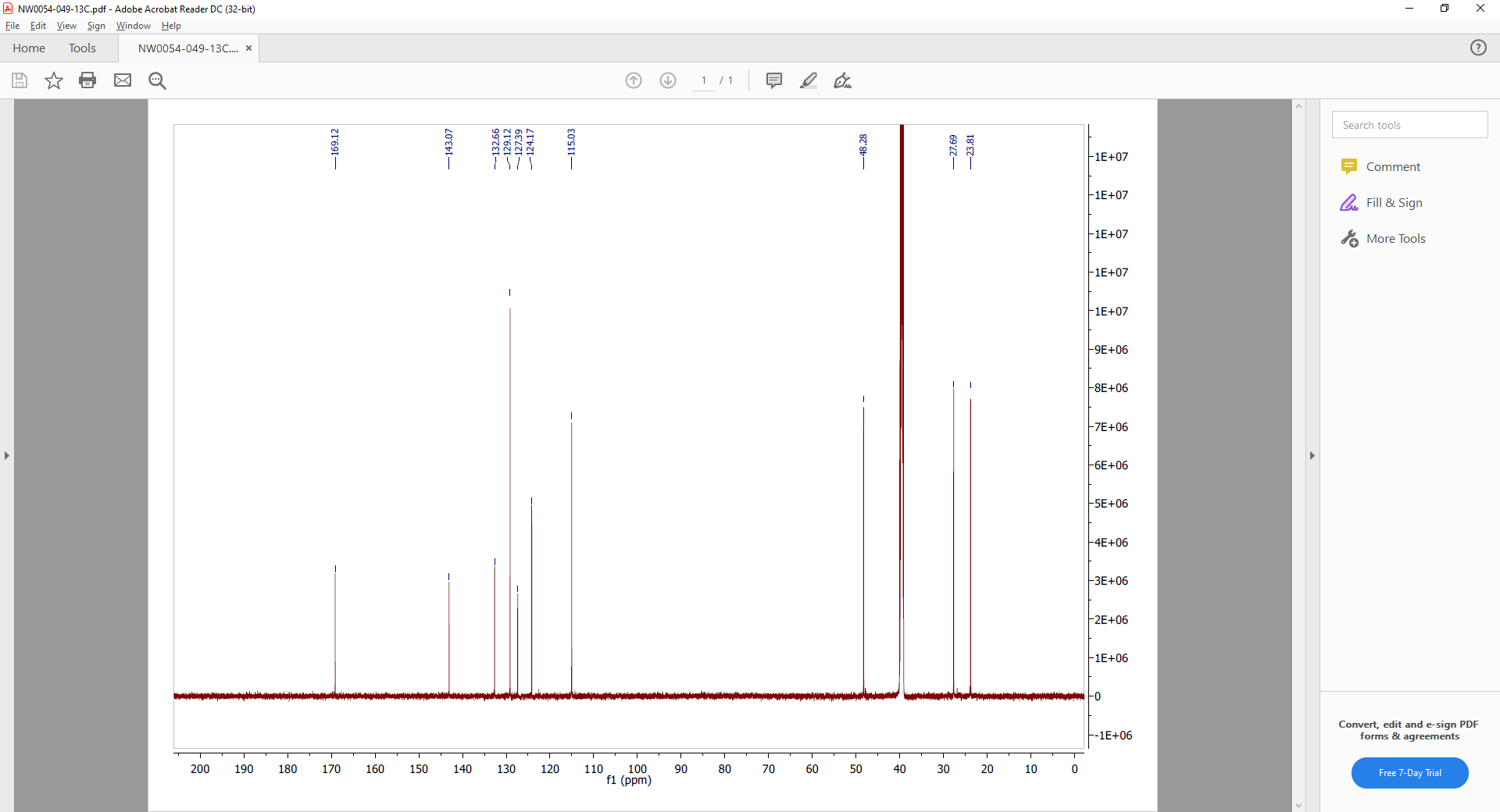


**Figure S3B**. ^13^C NMR (176 MHz, DMSO-*d_6_*) for **4w**.


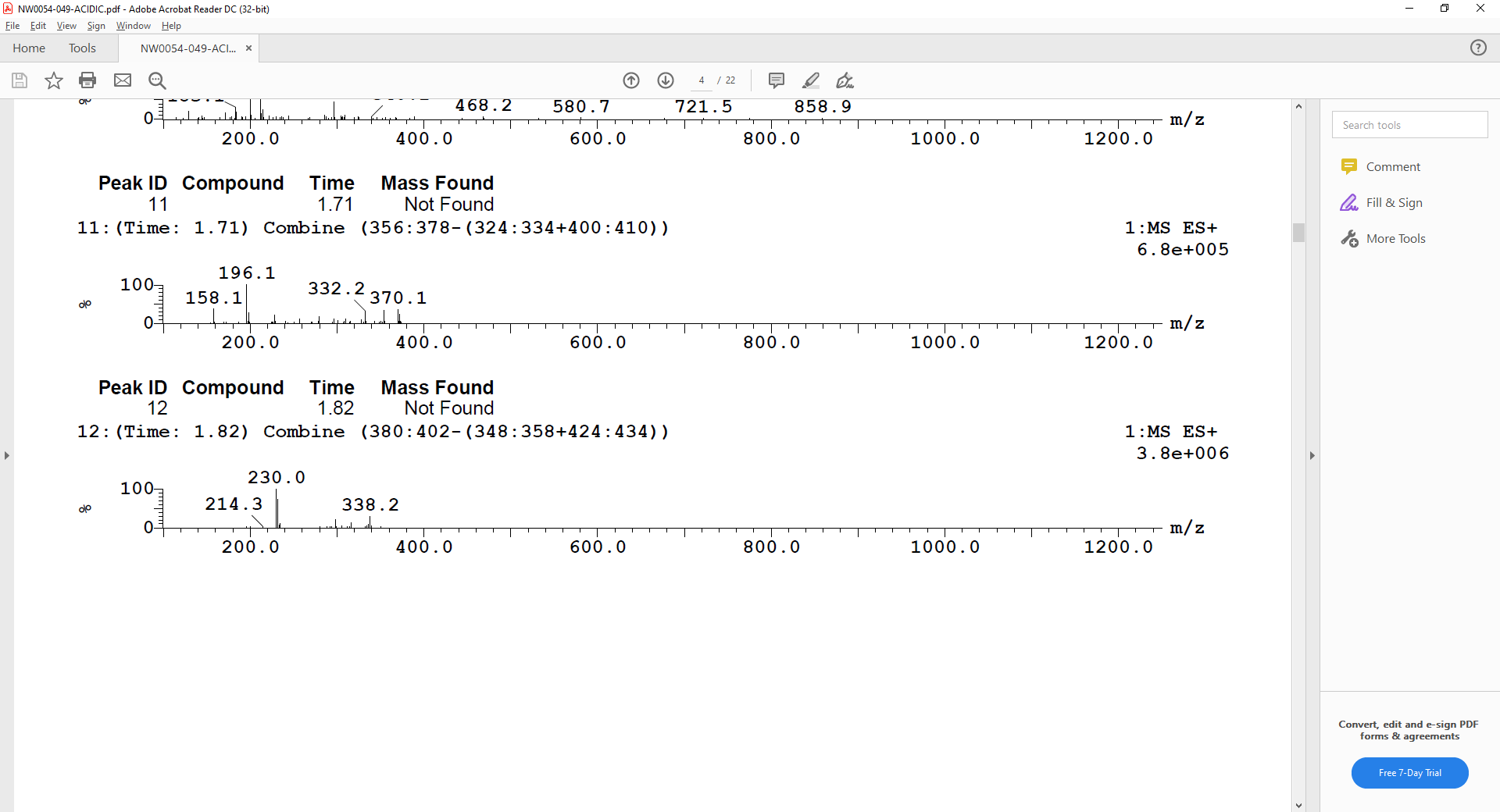


**Figure S3C**. LCMS (acidic) for **4w**.

Additional spectra and analytical data for compounds **3-5** will be made available upon reasonable request.

**Figure S4: Notum OPTS and TCF/LEF screening data concentration-response curves for 4w**

| 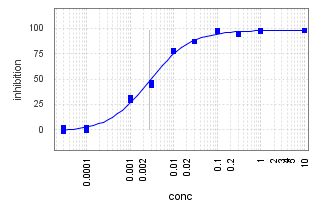 | 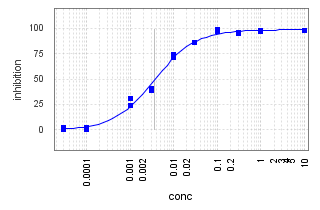 |
| --- | --- |
| IC_50_ 2.8 nM  [Ref: 155349] | IC_50_ 3.7 nM  [Ref: 155349] |
| 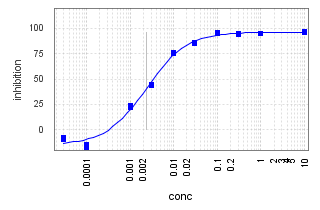 | 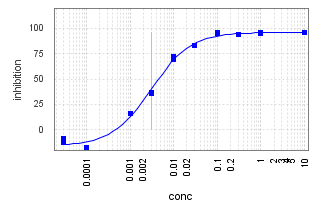 |
| IC_50_ 2.3 nM  [Ref: 155312] | IC_50_ 3.1 nM  [Ref: 155312] |

**Figure S4A.** Notum (81-451 C330S) OPTS data. Inhibition-concentration curves are representative, medium examples for **4w** (4 of 11 shown) from the larger data set for compounds **3-5**.

| 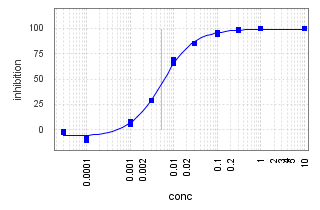 | 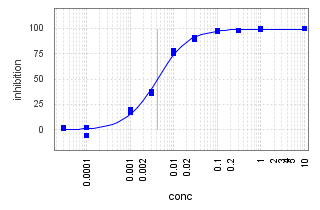 |
| --- | --- |
| IC_50_ 5.3 nM  [Ref: 159067] | IC_50_ 4.1 nM  [Ref: 159068] |
| 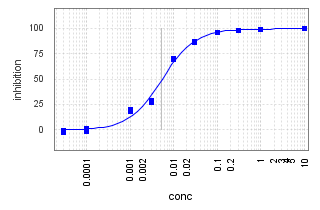 |  |
| IC_50_ 5.4 nM  [Ref: 159069] |  |

**Figure S4B.** Notum (full length) OPTS data. Inhibition-concentration curves for **4w** (3 of 3 shown) from the larger data set for compounds **3-5**.

| 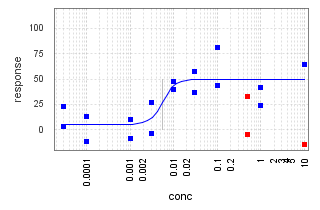 | 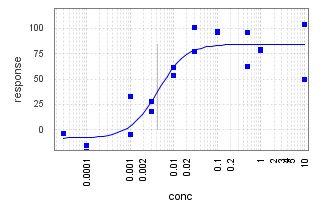 |
| --- | --- |
| EC_50_ 5.5 nM  [Ref: 156588] | EC_50_ 4.3 nM  [Ref: 156629] |
| 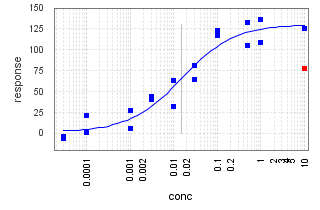 | 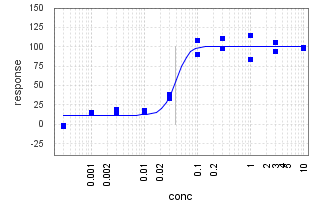 |
| EC_50_ 15.2 nM  [Ref: 156649] | EC_50_ 39.0 nM  [Ref: 157228] |
| 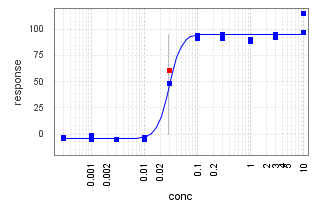 | 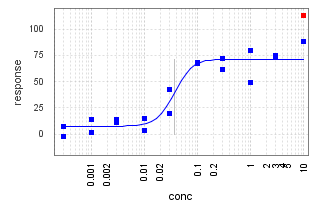 |
| EC_50_ 29.1 nM  [Ref: 157367] | EC_50_ 36.8 nM  [Ref: 157429] |

**Figure S4C.** Notum TCF/LEF reporter (Luciferase) data with WNT3A (200 ng/mL). Response-concentration curves are for **4w** (6 of 6 shown) from a larger data set for compounds **3-5**. Examples are from different screening runs.

| 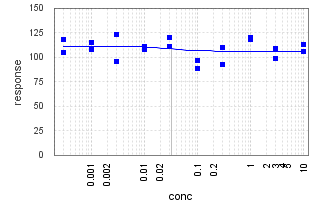 | 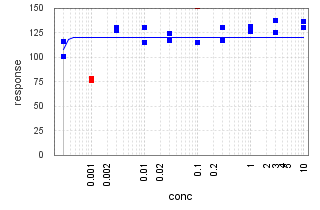 |
| --- | --- |
| [Ref: 157368] | [Ref: 156690] |

**Figure S4D.** Notum TCF/LEF reporter (Luciferase) data with WNT3A (200 ng/mL) in the *absence* of Notum. Representative response-concentration curves are for **4w** (2 of 6 shown) from a larger data set for compounds **3-5**. Examples are from different screening runs.

Additional inhibition-concentration curves for compounds **3-5** will be made available upon reasonable request.

**SM References**

1. Steadman, D.; Atkinson, B. N.; Zhao, Y.; Willis, N. J.; Frew, S.; Monaghan, A.; Patel, C.; Armstrong, E.; Costelloe, K.; Magno, L.; Bictash, M.; Jones, E. Y.; Fish, P. V.; Svensson, F. Virtual screening directly identifies new fragment-sized inhibitors of carboxylesterase Notum with nanomolar activity. *J. Med. Chem.* **2022,** *65*, 562-578.
2. Willis, N. J.; Mahy, W.; Sipthorp, J.; Zhao, Y.; Woodward, H.; Atkinson, B. N.; Bayle, E. D.; Svensson, F.; Frew, S.; Jeganathan, F.; Monaghan, A.; Benvegnu, S.; Jolly, S.; Vecchia, L.; Ruza, R. R.; Kjaer, S.; Howell, S.; Snijders, A.; Bictash, M.; Salinas, P. C.; Vincent, J-P.; Jones, E. Y.; Whiting, P.; Fish, P. V. Design of a potent, selective and brain penetrant inhibitor of Wnt-deactivating enzyme Notum by optimization of a crystallographic fragment hit. *J. Med. Chem.* **2022**, *65*, 7212-7230.
